# Near-infrared fatty acid molecular probe for image-guided surgery of glioblastoma

**DOI:** 10.1038/s44303-025-00077-z

**Published:** 2025-06-23

**Authors:** Meedie Ali, Pavlo Khodakivskyi, Ioannis Ntafoulis, Koen T. H. van der Kuil, Kranthi M. Panth, Arno Roos, Aleksey Yevtodiyenko, Kevin P. Francis, Zhenyu Gao, Martine L. M. Lamfers, Clemens W. G. M. Löwik, Laura Mezzanotte, Elena A. Goun

**Affiliations:** 1 Department of Radiology and Nuclear Medicine, Erasmus MC Cancer Institute, Erasums university Medical Center, Dr. Molewaterplein 40, Rotterdam, The Netherlands; 2 Department of Molecular Genetics, Erasmus MC Cancer Institute, Erasmus University Medical Center, Dr. Molewaterplein 40, Rotterdam, The Netherlands; 3 Department of Neurosurgery, Erasmus MC Cancer Institute, Brain Tumor Center, Erasmus University Medical Center, Dr. Molewaterplein 40, Rotterdam, The Netherlands; 4 Department of Chemistry, University of Missouri, 601 S College Ave, Columbia, Missouri 65211 USA; 5 Department of Neuroscience, Erasmus University Medical Center, Dr. Molewaterplein 40, Rotterdam, The Netherlands; 6 Veterinary Referral Centre Korte Akkeren, Emmastraat 29, Gouda, The Netherlands; 7 SwissLumix SARL, Batiment C, Lausanne 1015, Switzerland; 8 Department of Orthopaedic Surgery, The University of California, Los Angeles, 615 Charles E. Young Drive S, Los Angeles, California 90095 USA; 9 PerkinElmer, 68 Elm Street, Hopkinton, MA USA

**Keywords:** Cancer imaging, Fluorescent dyes, Fluorescence imaging, Brain imaging, Molecular imaging, Imaging techniques and agents

## Abstract

Metabolic reprogramming is considered a major driving factor in cancer growth and yet it remains challenging to monitor in vivo uptake of fatty acids, which are essential energy sources for many tumor types. Here, we report the development of a novel, long-chain fatty acid (FA), near-infrared (NIR) imaging reagent (FA-ICG) for real-time, non-invasive imaging of FA absorption in vitro and in vivo. Moreover, we demonstrate the application of the probe in image-guided cancer surgery, where precise assessment of tumor margins is paramount for removal. Specifically, we focus on glioblastoma (GBM), where FA metabolism plays a key role in progression and where there is a significant need for better intraoperative imaging. Here, we successfully demonstrate the application of the probe for NIR in vivo imaging in two different orthotopic models of GBM. In addition, we validate the uptake of the probe in companion dogs with mastocytomas, as these develop cancer with a similar pathology to humans. Our results demonstrate that the probe combines benefits from NIR imaging, such as high sensitivity, low autofluorescence, and deep tissue penetration, with specific tumor metabolism-based targeting and retention. Thus, it represents a promising candidate for a wide range of applications in the fields of metabolic imaging, drug development, and most notably for translation in image-guided surgery.

## Introduction

Exogenous fatty acids (FAs) are primarily sourced from diet and are key regulators of metabolic homeostasis and immune response^1,2^. Thus, their uptake is significantly altered in a wide range of pathological conditions including rheumatoid arthritis^3^, insulin resistance and obesity related disorders^4^, inflammatory bowel disease (IBD)^5^, asthma^6^, intestinal malabsorption^7^, neurodegenerative disease^8^, cardiovascular disease^4,9^, hepatosteatosis^10,11^ and lipotoxicity in liver, heart, and pancreatic β-cells^12–14^. Importantly, FA metabolism plays a key role in tumor metabolic reprogramming because tumor tissues heavily depend on FAs to sustain their rapid proliferation and to provide essential energy sources during conditions of metabolic stress^15–18^. In addition, FA metabolism is central for cancer immunity and controls important processes such as inflammation^15,19–21^. Thus, many transporters responsible for the uptake of FAs are significantly overexpressed in a wide range of human cancers and this overexpression is strongly associated with poor prognosis^22–30^. Moreover, the tumor cells possess an increased capacity to accumulate FAs into dynamic intracellular lipid droplets, especially under conditions of hypoxia or starvation^31,32^. Importantly, many recent studies have demonstrated that tumors also greatly depend on extracellular free FAs to survive and proliferate, and that de novo synthesis and exogenous FA uptake are equally important in driving cancer progression^4,7,8,12^.

In glioblastoma in particular, recent evidence suggests that malignant infiltrative cells, also classified as glioma stem-like cells (GSCs), rely both on exogenous FA uptake and de novo FA synthesis to generate energy^33–35^. Therefore, targeting FA transport pathways represents a promising strategy for the development of novel anticancer therapies^15,36–38^. Recently, many of such biological targets were discovered and tested by our group and others^15,36–42^. Thus, quantifying changes of exogenous FA fluxes is of critical importance for understanding the underlying mechanisms of human diseases, the development of novel effective treatments, and for drug discovery. In addition, FA metabolic reprogramming has been actively investigated as a target for oncologic imaging approaches^43^. Several non-radioactive methods have recently been developed for non-invasive quantification of FA uptake in cells and living animals^44,45^. In nuclear medicine, FA linked PET tracers, [^11^C]-palmitate and [^11^C]-acetate, have been developed to monitor de novo FA synthesis^46–48^. However, the short half-life of carbon-11 (*t*_½_ = 20.4 min) requires a cyclotron in-house to generate the radioactive isotope. Furthermore, the products are quickly metabolized^49^ leading to a reduced image quality and difficulties in detecting the synthetic derivatives. To overcome these drawbacks, ^18^F-labeled FA analogs (*t*_½_ = 109.8 min) have been developed, such as 14-(R,S)-[^18^F]-fluoro-6-thia-heptadecanoic acid (FTHA)^50^, 16-[^18^F]fluoro-4-thia-palmitate (FTP)^51^, and 18-[^18^F]fluoro-4-thia-oleate (FTO)^52,53^. Gallium-68 (*t*_½_ = 68.3 min) chelates conjugated to long-chain FAs were investigated as potential myocardial metabolic PET imaging agents^54,55^. Contrary to initial expectations, these probes demonstrated insufficient accumulation in the heart as well as low contrast ratios compared to non-targeted organs. Alternatively, β-Methyl [^125^I] iodophenyl-pentanedecanoic acid (^125^I-BMIPP) has been used as an adipose tissue imaging agent preclinically^56,57^, while its radio-isotopic analog, β-Methyl [^123^I] iodophenyl-pentadecanoic acid (^123^I-BMIPP), has been used widely as a non-invasive cardiac metabolism imaging agent for single-photon emission computed tomography (SPECT) in clinical studies^58^. Despite the significant potential of nuclear tracers, the use of ionizing radiation, high costs, and instrumental limitations in image-guided surgery have necessitated the development of alternative methods for in vivo imaging^59,60^.

Optical imaging is typically characterized by high sensitivity (10^−9^ to 10^−15^ mole), low cost, and long-term stability of optical reagents^61^. Recently, several novel optical reagents have been published for the study of FA uptake. For example, we have previously reported a bioluminescence-based probe for non-invasive FA uptake in vitro and in vivo^62,63^. While bioluminescence imaging is considered the most sensitive modality in preclinical imaging^64^, it has limited capacity for clinical translation^65^. In addition, several fluorescently labeled, long-chain FA probes were developed in the past that were successfully utilized for imaging of FA uptake in live cells, intracellular organelles such as peroxisomes^66^ and most notably for in vivo imaging of brown adipose tissue and cardiac metabolism^67,68^. However, no FA-based fluorescent probes were previously reported for non-invasive imaging of cancer metabolism in vivo. Importantly, many recent reports have revealed evidence that the metabolism of cancer cells in vitro can differ significantly from that of in vivo (animal) models of disease^69–71^ yet the progress of studying tumor metabolism in vivo is significantly hampered by the lack of efficient tools that allow non-invasive imaging and quantification of FA absorption.

In this work, a long-chain saturated FA, palmitic acid, has been covalently linked to indocyanine green (ICG) to form FA-ICG, a NIR imaging probe for FA metabolism. The clinically approved ICG dye is a NIR dye that enables deep tissue imaging, providing a high signal-to-noise ratio compared to fluorophores that absorb and emit light in the visible spectrum. Due to the favorable excitation and emission spectra, FA-ICG suffers only minimal absorption and scattering from biological tissues since the tissue autofluorescence is negligible in this window^72,73^. Taken together, these optical properties allow for a more specific signal that, in theory, should lead to improved tumor contrast ratio.

One of the new applications of the NIR optical probes is image-guided surgery of cancer, an exciting area of clinical science that allows surgeons to completely remove tumors while avoiding damage to healthy tissues^74,75^. Since surgery has a key role in the management of many types of human cancers, efficient tumor removal is known to result in much higher survival rates, better overall prognosis, and reduction of surgical complications. However, removal of certain tumors, such as glioblastomas, can be very challenging, as cancerous tissues can look very similar to normal brain. Thus, new imaging tools that allow precise visualization of tumor margins intraoperatively are urgently needed. The only optical agent that has been shown to improve long-term outcomes after a surgical intervention in oncological surgery is 5-aminolevulinic acid (5-ALA)^76^, which was approved for glioblastoma surgery by the U.S. Food and Drug Administration in 2017. The design of the probe relies on 5-ALA being metabolized into protoporphyrin IX (PpIX), which accumulates in cancer cells due to the low activity of an enzyme that metabolizes PpIX to heme (ferrochelatase). The resulting compound emits a fluorescent signal with two peaks at 635 nm and 710 nm. However, this imaging reagent has multiple drawbacks, such as significant photosensitivity and photobleaching. In addition, it requires excitation with blue light, which limits tissue penetration depth^77^ and requires the light in the surgical room to be dimmed to avoid confusing the signal from the probe with the light reflection^78^. Most importantly, the sensitivity of detecting PpIX is limited, and 5-ALA has therefore been criticized for its weak negative predictive value, i.e., the large proportion of non-fluorescing tissues containing (undetected) tumor cells. In addition, recent findings indicate that 5-ALA mostly accumulates in myeloid cells and tumor-associated macrophages^79^, making this probe less specific for highlighting the entire tumor margin. These significant limitations necessitated the attempt to use another well-known dye, free ICG, for image-guided surgery of brain cancer^80^. Despite its excellent NIR optical properties that are superior to 5-ALA, free ICG dye has other considerable drawbacks such as very short plasma half-life (3 min), high dose requirement for brain tumor imaging (5 mg/kg), and significant nonspecific signal from blood^81^.

To address an urgent need in the development of novel tools for image-guides surgery of cancer, we developed a probe that is based on the observation that many cancer types, including glioblastomas, exhibit significantly increased FA metabolism^82–84^. Thus, we reasoned that conjugation of a FA with the FDA-approved NIR dye ICG might result in specific accumulation of the probe in tumors, thereby allowing efficient intraoperative visualization of tumor margins. By combining the intrinsic low autofluorescence of ICG with the improved selectivity of its fatty acid targeting moiety, we hypothesize that this probe will provide a more specific uptake mechanism for glioblastoma cells.

In this work, we first validated the use of FA-ICG to track FA uptake in vitro in adipocytes and then in cancer cells. Our results indicated that the FA-ICG probe resembles the natural uptake of long-chain FAs. We then demonstrated its use for non-invasive imaging of brain tumors in two animal models of glioblastoma. Lastly, we successfully utilized the FA-ICG probe as a viable tool for image-guided surgical resection of cancer in companion dogs with mastocytoma that develop cancer with similar pathology to humans. Our results demonstrate that FA metabolism represents an excellent target for tumor imaging, leading to significantly enhanced uptake of the FA-ICG probe in tumors. The novel probe allows sensitive image-guided surgery using various imaging systems in different animal models of cancer, ultimately paving the way for clinical translation.

## Results

### Design, synthesis, and spectroscopic characterization of the FA-ICG probe

**Fig. 1:**
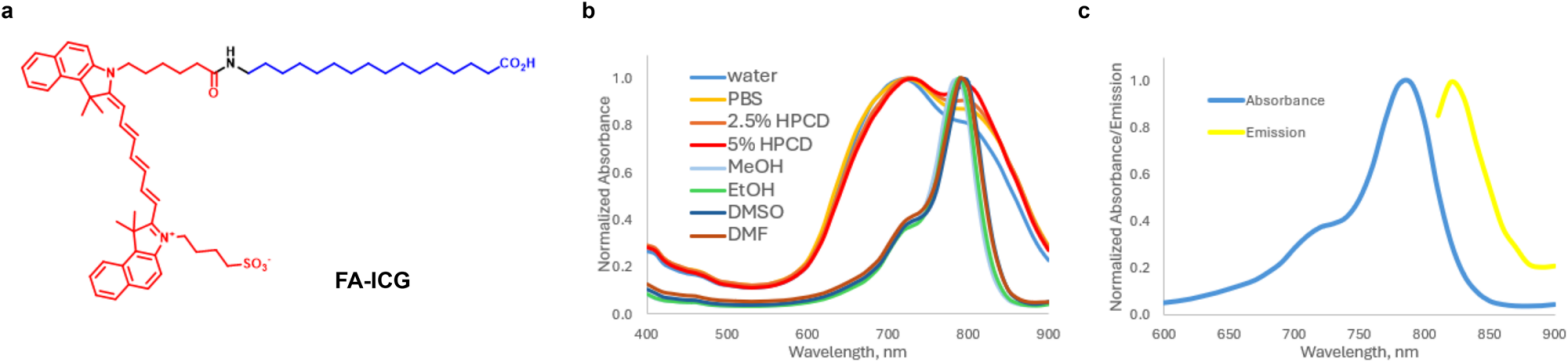
FA-ICG probe: structure and spectroscopic characterization. **a** Structure of FA-ICG probe, FA is shown in blue and ICG is shown in red. **b** Normalized absorbance of FA-ICG in various polar solvents (10 µM). **c** Fluorescence spectra of FA-ICG in methanol: absorbance (solid blue line) and emission at a fixed excitation wavelength 780 nm (solid yellow line). The synthesis of the probe is described in the supplementary information.

The structure of the FA-ICG probe is shown in Fig. 1a. The general concept of our design was to attach the optical imaging probe ICG to a long-chain FA via a covalent bond. The choice of the fluorescent dye was based on the wide clinical use of ICG dye as an FDA-approved drug for intraoperative evaluation of tissue perfusion, such as identification of neurovascular anatomy, ophthalmic structures, and sentinel lymph nodes^85,86^. Therefore, for the FA-ICG probe to be taken up by the same physiological transport process as natural FAs, we selected to attach the dye to palmitic acid. Previous reports on structure activity of FA transporter (FATP) substrate specificity describe the requirements of the carbon chain to be >10 carbons, even numbered, unbranched, and the carboxylic acid to be non-esterified^87^. In short, FA-ICG was synthesized by amide coupling between ICG-NHS activated ester^88^ and 16-aminohexadecanoic acid, which was obtained by catalytic hydrogenation of 16-azidohexadecanoic acid. Detailed synthetic procedures and characterization of intermediates and the FA-ICG probe are provided in the supplementary information (Scheme S1). Absorbance of FA-ICG in different conditions demonstrates that the probe has a peak at 780 nm and an additional peak at 720 nm (e.g., in PBS or water, Fig. 1b). Emission spectrum of FA-ICG was found to be comparable to that of ICG with the peak of emission at 820 nm (methanol, Fig. 1c). Since ICG dye is FDA-approved and widely used for many clinical applications, these spectroscopic characteristics make this probe compatible with the vast majority of intraoperative cameras and surgical microscopes^85^.

### Imaging and quantification of FA uptake in living cells using the FA-ICG probe

**Fig. 2:**
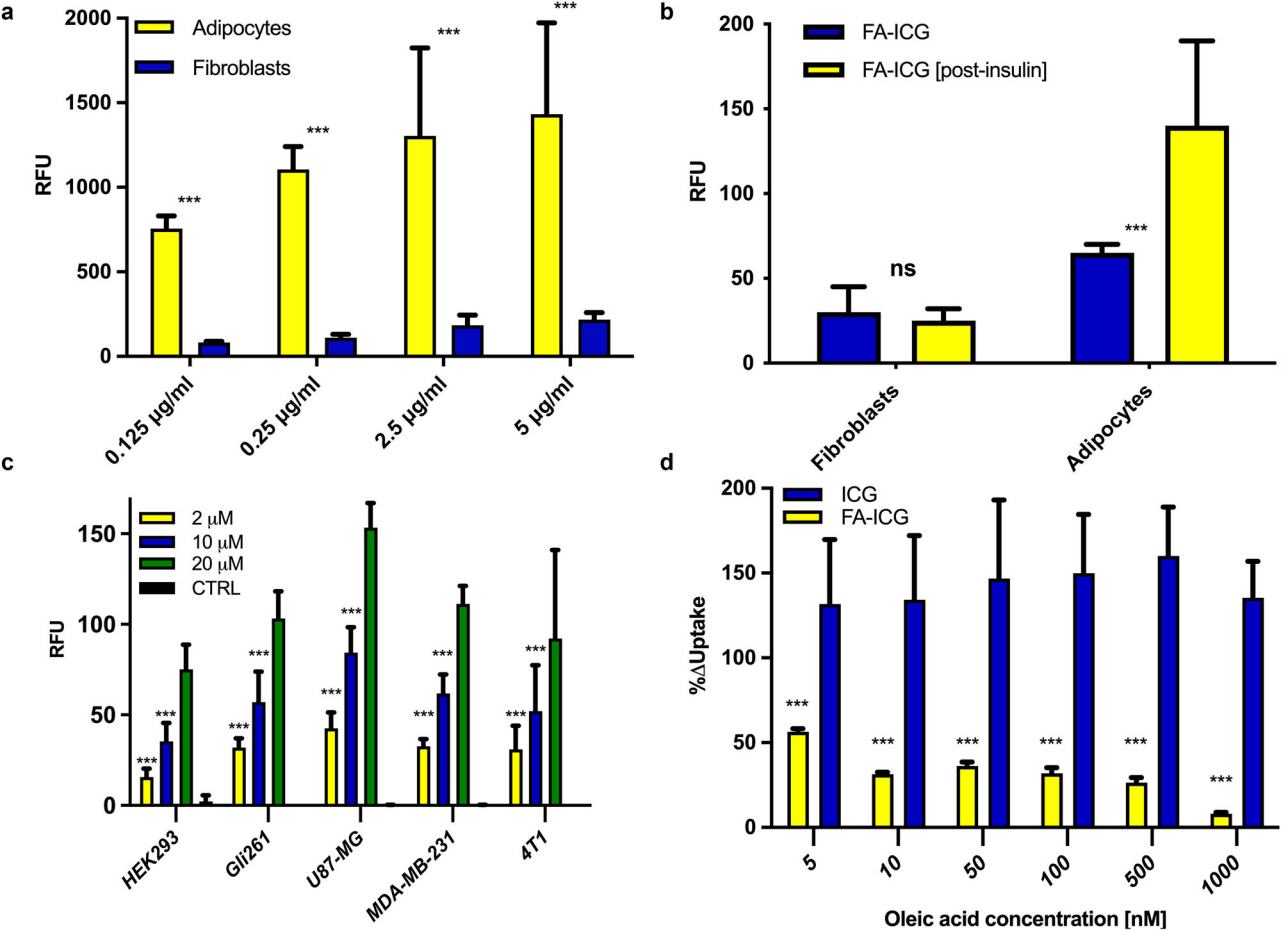
Imaging and quantification of FA uptake in living cells using the FA-ICG probe. **a** Dose-dependent uptake of FA-ICG resulting from 3T3-L1 adipocytes and fibroblasts. Adipocytes demonstrate an increase in FA-ICG uptake at higher concentrations, whereas fibroblasts demonstrate an early plateau in uptake. Comparison of the resulting signal from 3T3-L1 adipocytes and fibroblasts demonstrates much higher uptake rates of the FA probe by the differentiated adipocytes, in agreement with previous reports; **b** Insulin stimulation results in a significant increase in the uptake of FA-ICG in adipocytes, while uptake in fibroblasts remains unaffected. **c** Fluorescent signal resulting from incubation of different cancer cell lines exposed to increasing concentrations of FA-ICG (2, 10, and 20 µM). Control cells received uptake buffer without FA-ICG. Glioblastoma cell line U87-MG demonstrated the highest uptake of the FA-ICG probe. **d** Comparison of FA-ICG and free ICG signal with increasing concentrations of natural oleic acid in U87-MG cells. As expected, FA-ICG uptake was significantly decreased by oleic acid competition while the signal resulting from ICG control remained unchanged. Experiments were performed in triplicate. Error bars report on standard deviation. Statistical analyses were performed using a one-way ANOVA test followed by the Bonferroni-Dunn method for multiple mean comparison. Statistical significance was set at *p* <0.05 (*< 0.05, **<0.01, ***<0.001).

To determine whether the FA-ICG probe is taken up by the same physiological transport process as natural FAs, we compared the uptake of FA-ICG in 3T3-L1 cells that can be readily differentiated from a fibroblast precursor to a terminally differentiated adipocyte. Cells were seeded into clear multi-well plates, incubated with increasing concentrations of FA-ICG solution (0.125–5 µg/mL) in 0.1% BSA-HBSS buffer for 30 min, rinsed 3 times, and imaged using an Odyssey CLx scanner (LI-COR). As shown in Fig. 2a, 3T3 adipocytes demonstrate a dose-dependent increase in uptake rates in the concentration range from 0.125–5 µg/mL, whereas fibroblasts exhibit an early plateau in uptake. Importantly, comparison of the resulting signal from 3T3 adipocytes and fibroblasts demonstrates much higher uptake rates of the FA compound by the differentiated adipocytes (Fig. 2a), in agreement with previous reports based on radio- and BODIPY-labeled FAs^89^.

To further test whether FA-ICG uptake resembles the physiological uptake of natural FAs, we investigated the known stimulatory effect of insulin on FA uptake using the FA-ICG probe^90^. We stimulated 3T3-luc adipocytes and fibroblast cells with insulin after the starvation of cells. We observed that insulin stimulation results in significantly increased uptake of FA-ICG in adipocytes while fibroblasts remain unaffected (Fig. 2b). These findings are aligned with previously reported results^90–92^. After demonstrating that the uptake of FA-ICG depends on FA transport through the cell membrane, we hypothesized that such uptake should also occur in malignant tumor cells that display an altered FA metabolism. Thus, we tested uptake of FA-ICG in several cancer cell lines and found that glioblastoma cells take up the FA-ICG probe at very high concentrations (U87-MG, Fig. 2c). Thus, we decided to use this cell line to investigate whether the signal from the FA-ICG probe could be inhibited by an excess of unlabeled oleic acid, a natural substrate for FATP. The results shown in Fig. 2d demonstrate strong inhibition of FA-ICG uptake by competition with natural, unlabeled oleic acid. As expected, the resulting decrease in signal is proportional to increased concentrations of oleic acid. Importantly, this effect was not observed in cells exposed to standard (unconjugated) ICG, which was used as a negative control. Altogether, these data further confirm that the specific FA-ICG uptake resembles the uptake of natural FA transport inside the living cell.

### Imaging and co-localization of FA-ICG uptake in orthotopic glioblastoma xenograft model (U87-MG) and patient-derived xenograft (PDX) models of glioblastoma

**Fig. 3:**
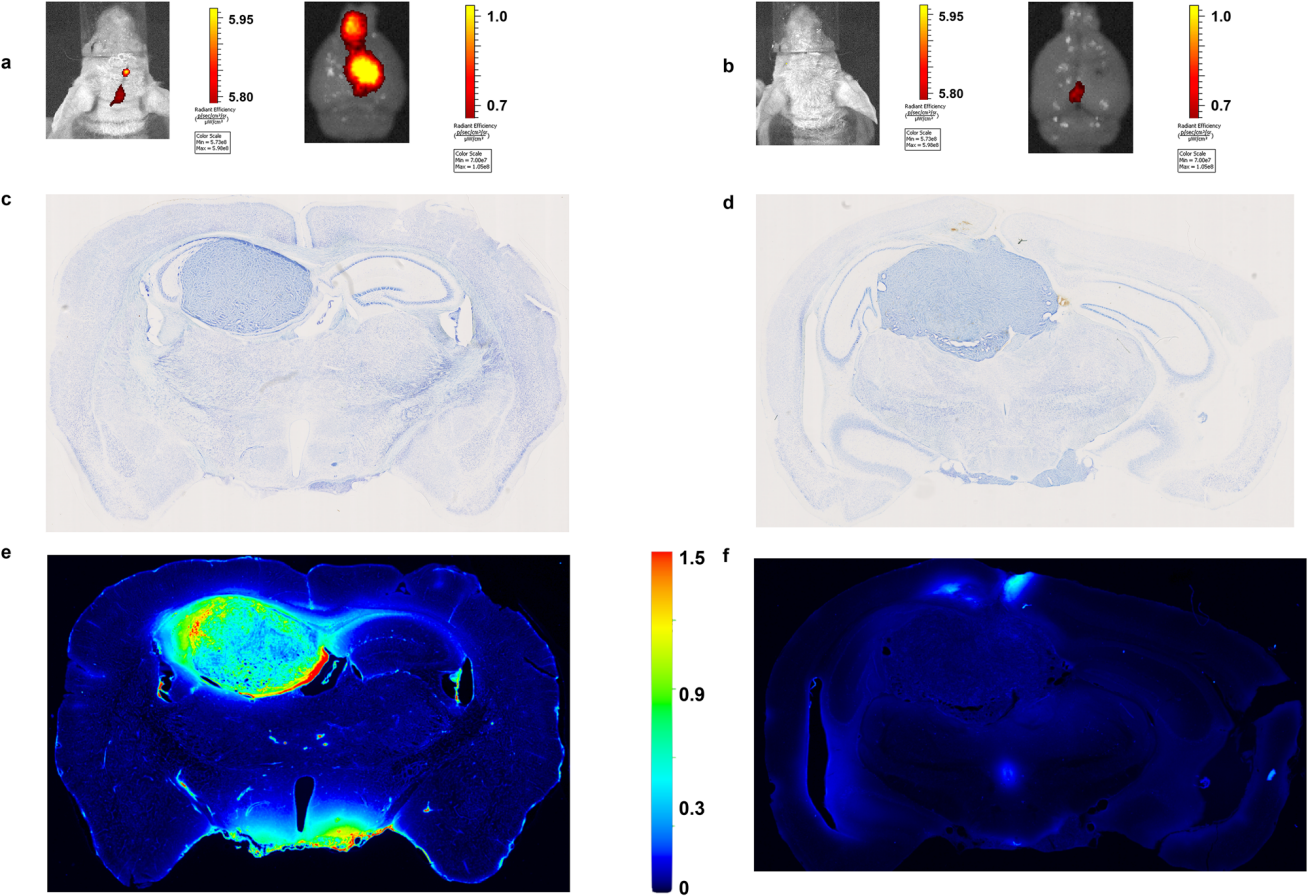
In- and ex vivo comparison of FA-ICG (vs ICG) accumulation in orthotopic U87-MG glioblastoma xenograft model. **a**, **b** In- and ex vivo fluorescence accumulation of FA-ICG (**a**) compared to fluorescence accumulation of ICG (**b**) at 8 h after drug administration on IVIS® Spectrum. Higher accumulation of FA-ICG (compared to ICG) is demonstrated on the right side of the head and in the right (tumor-bearing) brain hemisphere. **c**, **d** Coronal thionine-stained brain sections demonstrate tumor presence in the right cerebral hemisphere in two different mouse sections (in dark violet). **e** NIR imaging demonstrates increased NIR fluorescence signal in the same brain slice on flatbed imaging (**c**, **e** show same slice), tumor presence co-localizes with NIR signal **f** Limited NIR fluorescence signal is observed in the ICG administered mouse, albeit slightly more in the tumor than in surrounding brain as compared with thionine staining (**d**), **d**, **f** show same slice. The sample size per experimental group described is *n* = 3. Error bars report on standard deviation. Statistical analyses were performed using a one-way ANOVA test followed by the Bonferroni–Dunn method for multiple mean comparison. Statistical significance was set at *p* < 0.05 (*< 0.05, **<0.01, ***<0.001).

Next, we investigated the uptake of FA-ICG in an orthotopic glioblastoma xenograft model (U87-MG). As shown in Fig. 3, supplementary Figs. S1–S6 and supplementary Movie 1, we observed high accumulation of the probe in the brain tumors when imaged by IVIS Spectrum and FMT-(CT) fluorescence imaging systems.

Most notably, we compared the fluorescence signal after i.v. administration of an equimolar dose of FA-ICG to that of ICG. Here, we observed an average radiance of approximately 2.2 times higher in FA-ICG administered mice than in ICG administered mice in- and ex vivo (Fig. 3 and supplementary Fig. 6). Next, the brain tissue slices were imaged on a NIR scanner, and the NIR signal demonstrated excellent co-localization of the tumor tissue and FA-ICG signal as shown in Fig. 3c–f. This signal (of FA-ICG) was found to be demonstrably higher than in ICG-treated mice, as shown.

Additional imaging experiments using FMT(-CT) further confirmed the accumulation of the probe in U87-MG-luc brain tumors (Supplementary Figs. S1–S3 and SI Movie 1). Here, three-dimensional co-localization of NIR and BLI signals was observed and reconstructed in 3D. In addition, tumor-to-background ratio (TBR) measured on IVIS imaging and determined by drawing a region of interest in the right side of the head (tumor-bearing hemisphere) compared to the left side of the head (background or control hemisphere), was shown to increase over time, reaching a considerably higher TBR at 12–24 h post-injection (Supplementary Fig. S4b). Moreover, we investigated whole body biodistribution of the probe over time and found that the probe was largely retained by the liver and kidneys (Supplementary Fig. S4 and ex vivo images of organs in Supplementary Fig. S5).

Since the accumulation in the brain was of particular interest to us, we divided the brain into a right ‘tumor-bearing’ (hemisphere) and a left ‘non-tumor bearing’ hemisphere, which were studied separately to compare the fluorescent signal in the separate hemispheres. While we observed that the initial retention of the probe was similar (at 8 h), at 12 and 24 h the retention was found approximately 2–3 times higher in the tumor-bearing (right) hemisphere (when compared to the non-tumor bearing hemisphere, Supplementary Fig. S4c, d). This observation was also confirmed by an improvement of in vivo TBR demonstrated over time (Supplementary Fig. S4b). Additional histological tumor confirmation is provided in Supplementary Fig. S7.

Following these results, we investigated the application of FA-ICG as a preclinical imaging agent in a patient-derived xenograft (PDX) model of glioblastoma (Supplementary Fig. S8). Here, we demonstrated that the probe could be successfully used for imaging of tumor growth at different time points in multiple mice (Supplementary Fig. S8a, b). This finding is of importance for preclinical research since PDX models are characterized by an unpredictable growth rate, while the introduction of optical imaging reporter genes such as luciferase is known to result in tumor phenotypic alterations^92^. Thus, these animal models would benefit from in vivo monitoring by non-invasive fluorescence imaging.

### Preclinical evaluation of FA-ICG as a novel probe for NIR image-guided cancer surgery

**Fig. 4:**
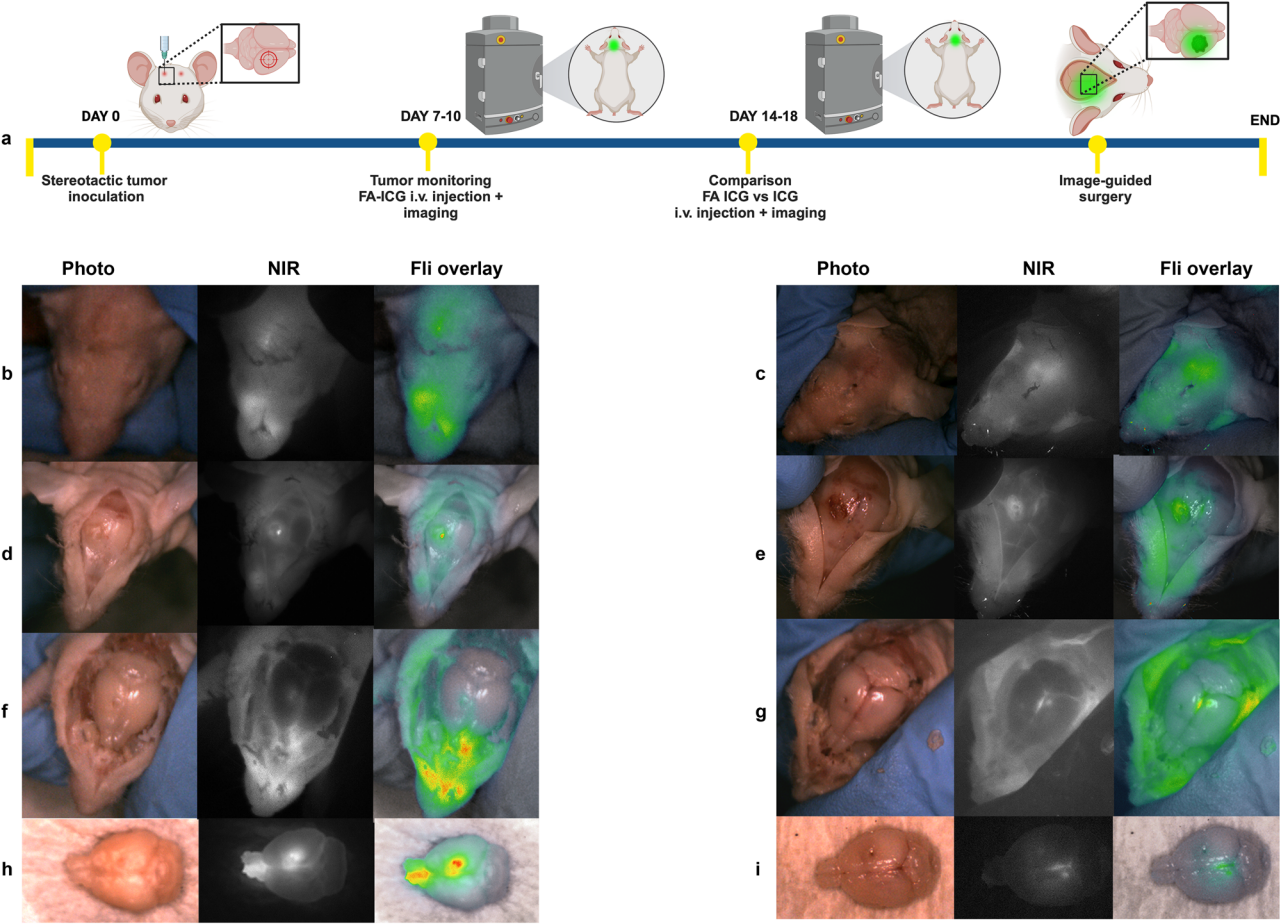
NIR image-guided surgery of U87-MG glioblastoma tumor-bearing mice (FA-ICG vs ICG). Intraoperative fluorescence imaging demonstrates accumulation of FA-ICG probe, left panel (**b**, **d**, **f**, **h**), when compared to ICG, right panel (**c**, **e**, **g**, **i**), on QUEST Spectrum® 2 image-guided surgery camera. **a** Schematic representation of the timeline of the experiment. **b**, **c** Transcutaneous fluorescence imaging demonstrates diffuse fluorescence signal in FA-ICG and ICG administered mice. **d**, **e** Transcranial fluorescence imaging demonstrates localized fluorescence signal (point or dot-shaped) in FA-ICG administered mouse (**d**) while more diffuse signal is observed in case of ICG (**e**). Notably, the brightfield image (**e**) shows more hemorrhage below the skull (and not clear tumor). **f**, **g** Higher intraparenchymal NIR signal (as shown in **f** and **h**) is demonstrated in the right cerebral hemisphere for FA-ICG, while in the ICG administered mouse (**g** and **i**) signal is observed in the middle (and the hemorrhage below the skull disappears). **h**, **i** Ex vivo imaging of the brain demonstrates considerably higher signal in the FA-ICG administered mouse than in the ICG administered mouse. The sample size per experimental group described is *n* = 3.

**Fig. 5:**
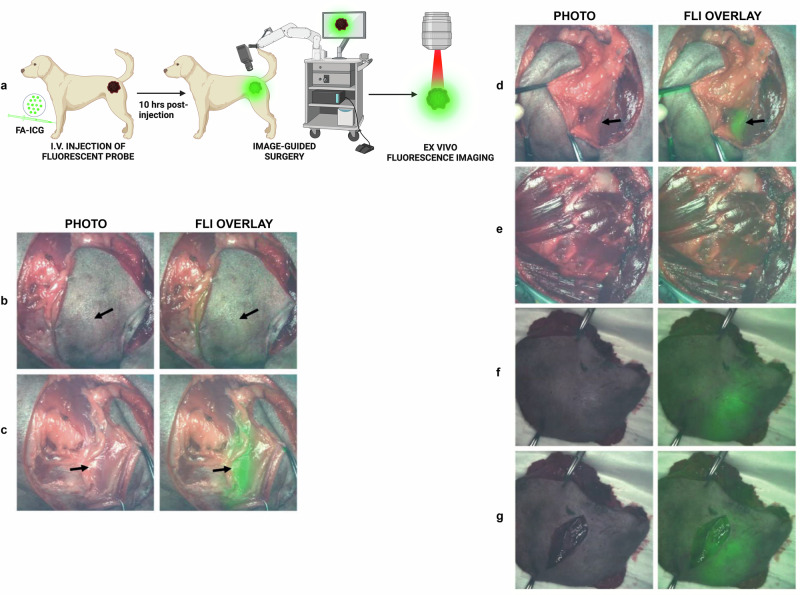
NIR image-guided surgery of canine mastocytomas using FA-ICG. A dog with a symptomatic mastocytoma located in the left upper hind leg was operated under NIR surgical camera guidance (PerkinElmer™, Solaris). **a** Schematic representation of fluorescence-guided surgery 10 h after administration of FA-ICG probe**. b** Intraoperative widefield and NIR fluorescence overlay of mastocytoma tumor demonstrates transcutaneous fluorescence signal of tumor (with skin removed around tumor) at 10 h post i.v. injection of 0.3 mg/kg FA-ICG imaging reagent. **c** Left tumor margin is exposed, demonstrating enhanced fluorescence signal from the tumor. **d** Lower, contralateral, right tumor margin is exposed, demonstrating fluorescence signal contralaterally. **e** Wound bed after resection demonstrates minimal remaining fluorescence signal. **f** Ex vivo fluorescence imaging of excised tumor with intact skin. **g** Ex vivo fluorescence imaging of excised tumor with open wound. (Arrows indicate tumor). Sample size of companion dogs included is *n* = 2. Surgery was performed by veterinarian Dr. Arno Roos (Veterinair Verwijscentrum Gouda, the Netherlands).

To demonstrate the feasibility of FA-ICG as a contrast agent for NIR image-guided surgery, we performed surgical operations on mice bearing orthotopic U87-MG glioblastoma tumors. Since the probe is based on clinically approved ICG dye, we utilized a standard (NIR) camera that has been clinically approved as instrument for use in the operating room with ICG (QUEST Spectrum**®** 2, also shown in Supplementary Fig. S9a). Using a similar setup as described above, we administered either the FA-ICG probe or the control (ICG) at 14–18 days after tumor cell inoculation. The experimental scheme is shown in Fig. 4a. Our results indicate that the FA-ICG probe is successful in imaging of tumor mass in the brain during the surgical procedure (Fig. 4 and SI Movie S2). Additionally, we found a considerably higher fluorescence signal in the brains of FA-ICG-treated mice than in the ICG-treated mice, as shown in Fig. 4h (compared to Fig. 4i) and Supplementary Fig. S6. These findings led us to further investigate the application of the probe in a surgical procedure in a companion dog with a symptomatic mastocytoma referred to a veterinary clinic for cancer removal. After i.v. injection of 0.3 mg/kg of FA-ICG, NIR image-guided surgery was successfully performed on a canine mastocytoma 10 h post-injection of the probe using an open-air NIR surgical camera (as depicted in supplementary Fig. S9b). The representative images shown in Fig. 5 and SI Movie 3, demonstrate consequent phases of the image-guided surgical operation and clearly demonstrate superimposed NIR signal in the tumor and in the subcutaneous fat pad.

## Discussion

FAs play a central role in the physiological metabolism of cells, and their aberrant uptake is involved in a wide range of human pathologies. Most notably, FA metabolism plays a key role in tumor metabolic reprogramming as a major source of energy needed to sustain the rapid proliferation of cancer cells^17,18^. Therefore, recent studies suggest that targeting FA transport pathways represents a promising strategy for the development of anti-cancer therapies^15,36–38^. Moreover, cancer (stem) cells are hypothesized to express alterations in metabolism to drive metastasis and evade the immune response^82,83^. Thus, a deeper understanding of fatty acid (FA) metabolism could offer critical insights into the underlying biology of cancer progression and reveal novel therapeutic targets, thereby leading to the development of more effective treatment strategies.

In the current study, we designed a novel probe where a long-chain FA is covalently linked to ICG (FA-ICG), a NIR dye that enables deep tissue imaging, providing a high signal-to-noise ratio. Importantly, this dye is FDA approved and widely used in clinical settings to assess tissue perfusion, and its spectral characteristics are ideally compatible with the vast majority of existing intraoperative cameras and surgical microscopes^85,86^. Since there is an urgent need for the development of novel imaging probes for image-guided surgery of cancer, we reasoned that conjugation of a FA to ICG might result in significant accumulation of the probe in tumors and would allow efficient intraoperative visualization of tumor margins. As mentioned above, this is of particular importance for efficient surgical removal of (malignant) brain tumors, where maximal removal of the tumor yet minimal damage to surrounding tissues is required^74,75^.

First, we successfully demonstrated that the uptake of FA-ICG probe resembles that of natural FAs by measuring the resulting signal from fibroblasts and differentiated adipocytes with and without insulin stimulation. Our results were fully aligned with previously reported data and indicate that FA-ICG uptake resembles the physiological uptake of natural FAs^62,90,91^. Next, we investigated the uptake of the FA-ICG probe in various cancer cell lines. We observed that glioblastoma cells take up the FA-ICG probe at very high concentrations (U87-MG). To further investigate the specificity of the signal from the FA-ICG probe, we performed a competition experiment where cells were incubated with FA-ICG in the presence of an excess of unlabeled oleic acid, a natural substrate for FATP. Altogether, our in vitro data confirm that the uptake of the FA-ICG probe represents the uptake of natural FAs in adipocytes and cancer cells.

We further confirmed these findings in orthotopic glioblastoma models, where enhanced uptake of FA-ICG was successfully demonstrated in the tumors and was retained over time, and its retention was superior to that of ICG. Additionally, we demonstrated that the probe could be utilized for imaging PDX tumors. This finding is of high relevance for preclinical research because these models are typically characterized by an unpredictable growth pattern and low tumor implantation rates. Thus, monitoring of tumor status by sensitive, non-invasive in vivo fluorescence imaging would be of high value as the introduction of optical imaging of reporter genes is known to result in tumor phenotypic alterations^92^. Next, we investigated whether the tumor targeting capabilities of FA-ICG in combination with the advantages of NIR imaging might enable application of this probe for image-guided surgery.

Currently, different targeting mechanisms for fluorescent dyes are studied in surgery, such as antibodies, enzyme-cleavable linkers, nanoparticles, and small peptides^93^. Although few targeted agents have been clinically approved until now, OTL-38 and LUM015 being the only notable exceptions^94,95^, ICG itself has been studied extensively in patients. Even though its use in general and oncological surgery is widespread, its application in brain tumor surgery has been very limited^96^. The only notable utilization of ICG has been the ‘second window ICG’ (or SWIG) trial in which patients were operated 24–48 h after high-dose administration of ICG^97^. This approach led to an improved negative predictive value over 5-ALA, widely considered to be the clinical standard in glioblastoma surgery^98^. However, nonspecific accumulation of ICG in vasculature and the short serum half-life, resulting in dose-related toxicities, remain the limiting factors for its further clinical translation for glioblastoma surgery.

In addition to SWIG, several recent reports also demonstrated visualization of ICG in the NIR II optical window (900 nm–1800 nm) in the (pre)clinical setting^99,100^, where it has been shown to improve tumor enhancement through dura and nontumorous cortical matter in patients^101^. Moreover, ICG has also been studied in multiple cancers with fluorescence lifetime imaging (FLT)^102^, where it demonstrated high specificity (of over 97%) for tumor cells over normal tissue, including blood vessels, independent of imaging system, dose, or time of injection. Since the design of our novel probe is based on ICG, our findings support further investigations of FA-ICG in the context of these novel approaches (SWIG and NIR II).

Taken together, our findings provide a strong basis for further translation of FA-ICG in image-guided cancer surgery. Our current efforts are directed at studying the pharmacological properties and safety in various formulations to initiate, ultimately, a phase I clinical trial in glioblastoma patients.

## Methods

### Probe synthesis and absorbance, and emission spectra

FA-ICG probe was synthesized by amide coupling between ICG-NHS activated ester and 16-aminohexadecanoic acid, prepared by catalytic hydrogenation of 16-azidohexadecanoic acid. Detailed synthetic procedures and characterization of intermediates and the probe have been provided in the supplementary text. Absorbance and emission spectra of the probe were determined in different solvents (water, PBS, DMSO, DMF, ethanol; methanol; aqueous solution of hydroxypropyl-β-cyclodextrin (HPCD).

### Cell cultures

3T3-L1 cells were obtained from ATCC. U87-MG, 4T1, MCF-7, MDA-MB-231, GL-261, and HEK293 cells were grown in Dulbecco’s Modified Eagle Medium (DMEM) containing 10% FBS and 1% penicillin/streptomycin. Patient-derived cell line (glioma stem cell culture), GS607, was established from the patient’ tumor tissue by the Department of Neurosurgery (Erasmus University Medical Center) after receiving patients’ informed consent and in accordance with protocols approved by the institutional review board. GSCs were cultured in serum-free (NS) medium containing, in short, Dulbecco’s modified Eagle’s medium-F12 with 1% penicillin/streptomycin, B27 (Invitrogen), human epidermal growth factor (5 µg/mL), human basic fibroblast growth factor (5 µg/mL; both from Tebu-Bio), and heparin (5 mg/mL; Sigma-Aldrich), grown on 1:100 diluted growth factor-reduced extracellular matrix coating (Cultrex, PathClear). All cultures were routinely tested for Mycoplasma.

### 3T3-L1 differentiation into adipocytes

3T3-L1 fibroblasts were grown in Dulbecco’s Modified Eagle Medium (DMEM) containing 4.5 g/L glucose, 10% fetal calf serum, and 1% penicillin/streptomycin. Differentiated cells were generated by treating fibroblasts 48 h post-confluency with DMEM containing 10% fetal bovine serum (FBS) and 1% penicillin/streptomycin, 1 µg/mL insulin, 0.25 µM dexamethasone, 0.5 mM IBMX, and 2 µM rosiglitazone (DM1-R medium) for 48 h. Then DM1-R medium was replaced by DMEM containing 10% FBS and 1% penicillin/streptomycin, supplemented with 1 µg/mL insulin (DM2) for 48 h, followed by maintenance in DMEM containing 10% FBS and 1% penicillin/streptomycin (DMS). Differentiated adipocytes were used between day 8 and day 12 after initiating the differentiation with DM1-R.

### FA-ICG uptake experiments in 3T3-L1 cells

3T3-L1 fibroblasts were seeded in 6-well plates with cellar bottom (Corning®, Flat Bottom, with lid, Sterile, Product #3335) at a density of 3000 cells/cm^2^ in DMEM (4.5 g/L glucose) containing 10% fetal calf serum and 1% penicillin/streptomycin. Differentiation into adipocytes was performed as described above. FA-ICG was dissolved in DMSO to get 10 mM or 50 mM stock solutions and was stored under nitrogen in the dark at −20 °C. Prior to the experiments, the stock solutions were diluted in 0.1% FA-free BSA in HBSS (with Ca^2+^ and Mg^2+^) to get the final uptake solutions, unless otherwise noted. Sodium oleate was dissolved in Millipore water to get a 100 mM stock solution, and aliquots were stored under nitrogen in the dark at −20 °C. For the dose-response experiment, 3T3-L1 fibroblasts and adipocytes grown in 96-well plates were serum-starved in FBS-free DMEM (4.5 g/L glucose) for 1 h before the assay and then incubated with FA-ICG solutions (0.125–5 µg/mL) in 0.1% FA-free BSA-HBSS for 30 min. Following the incubation, the cells were rinsed with HBSS containing 0.1% FA-free BSA and then washed three times with 0.5% FA-free BSA-HBSS for 10 min while keeping the cells on ice. After washing the cells, detection of fluorescent signal was performed using the Odyssey CLx scanner (LI-COR). For the experiment investigating the insulin dependence of uptake, adipocytes and fibroblasts were starved, and insulin was added to the uptake buffer at a concentration of 1 µg/mL.

### FA-ICG uptake assay in cancer cells

On day 0, cells were seeded in a black, clear-bottom 96-well plate (1 × 10^4^ cells) 24 h before the experiment. On day 1, after starvation in FBS-free DMEM for 30 min, the cells were incubated with either ICG or FA-ICG (1–20 µM) in HBSS containing 0.1% FA-free BSA for 3 h at 37 °C and were then washed three times with 0.5% FA-free BSA-HBSS for 10 min on ice. Detection of fluorescent signal was performed using an Odyssey CLx Scanner (LI-COR).

### FA-ICG competition assay in cancer cells

For the competition assay of FA-ICG and ICG, cells were seeded in a black, clear-bottom 96-well plate 24 h before the experiment. After starvation in FBS-free DMEM for 30 h, the cells were incubated with FA-ICG or ICG (1–20 µM for each probe) and increasing amounts of oleic acid (5–1000 nM) in HBSS containing 0.1% FA-free BSA for 25 min at 37 °C, and then the cells were processed using the protocol as described above for the dose-response experiment in 3T3-L1 cells.

### Animal welfare

Mice were monitored daily for signs of persistent abnormal behavior and/or breathing, over 20% weight loss, or development of neurological symptoms such as convulsions, seizures, and/or hemiparesis. When humane endpoints were reached, mice were euthanized using CO_2_. Animals were housed and treated in accordance with the animal protocols approved by the Ethical Committee of the Animal Welfare Body of Erasmus Medical Center Rotterdam, the Netherlands.

### In vivo small-animal imaging

In vivo imaging of mice was performed (at excitation 745 nm and emission 820 nm) using the IVIS Spectrum imaging system (PerkinElmer) and FMT2500 LX Fluorescence Tomographic Imaging system (PerkinElmer). Photon fluxes of regions of interest were calculated with the IVIS Living Image software or ImageJ.

### Fluorescence imaging and biodistribution in orthotopic U87-MG glioblastoma mouse model

U87-MG cells were orthotopically injected in immunodeficient NMRI nude mice (6–7 weeks old) in the right cerebral hemisphere (striatum) (2 × 10^5^ cells per mouse in 5 µL PBS) as described previously^103^. Fluorescence imaging was performed at 7–10 and 14–18 days after injection of tumor cells. Prior to imaging, mice were intravenously administered with FA-ICG. After injection, in vivo fluorescence imaging was performed under anesthesia with 2% isoflurane in oxygen. Animals were assigned to different groups by random allocation (imaging time point: 8-, 12-, and 24 h), different groups of mice (*n* = 4 per group) were sacrificed, organs were collected, and snap frozen. Subsequently, organs were homogenized and used to detect the relative fluorescence in homogenized tissue at the near-infrared Odyssey CLx scanner (at 800 nm) in 96-well black plates with clear bottom. A standard curve of FA-ICG was prepared in saline to relate the fluorescence signal in tissue to the fluorescence signal of drug doses. Additionally, brains were fixed in paraformaldehyde (PFA 4%) solution and embedded in paraffin. After paraffin embedding, brains were cut in 4 µm slices and stained using haematoxylin and eosin (H&E). Tumor (glioblastoma) presence was confirmed by a neuropathologist.

### Intraoperative- and ex vivo NIR fluorescence imaging of FA-ICG and ICG in orthotopic U87-MG glioblastoma mouse model

At days 14 to 18 after tumor inoculation, U87-MG orthotopic tumor-bearing mice were administered i.v. FA-ICG or ICG in equimolar doses (25 nmol/30 g, 250 µM, solution in 0.1% FA-free BSA in PBS). Eight hours after drug administration, IVIS imaging was performed. Subsequently, mice were euthanized by intracardiac perfusion with PFA 4%. After the mice were imaged, and brains were carefully removed under intraoperative guidance of the QUEST Spectrum® 2 camera. After which, the brains were fixed in PFA 4% and embedded in sucrose, so that afterwards the brains could be cut in 50 µm slices for ex vivo NIR fluorescence imaging on the ODYSSEY M scanner (LI-COR) at 800 nm with high resolution (5 µm), followed by the same-slice (histo)pathological staining in thionine for tumor-fluorescence co-localization.

### Fluorescence imaging in orthotopic PDX glioblastoma mouse model

Patient-derived glioblastoma cells (glioma stem-like cell line, GS607, passage number ranging from 8–10) were collected from patients and cultured as established by the Department of Neurosurgery (Erasmus University Medical Center), mentioned above. In the animal experiment, cells were orthotopically injected in immunodeficient non-obese diabetic severe combined immunodeficient gamma null (NSG) female mice (6–7 weeks old) in the right cerebral hemisphere (right striatum) (2.5 × 10^5^ cells per mouse in 5 µL PBS) as described in earlier work^104^. Starting 12 days after tumor inoculation, mice were intravenously administered with FA-ICG (20 nmol/25 g, 100 µM, solution in 0.1% FA-free BSA in PBS) and imaged. Fluorescence imaging was performed 24 h after administration of the probe, for which mice were shaved on the head, and imaging was performed on the IVIS imaging system using the excitation and emission spectra mentioned above.

### Intraoperative NIR fluorescence imaging of FA-ICG in companion dogs

Dogs with a symptomatic mastocytoma were operated on by (oncological) veterinary surgeon Dr. A. Roos (Veterinary Referral Center Gouda, the Netherlands). After informed consent was obtained from the owner and the dog was determined to be fit for surgery, the dog was included. Ten hours prior to surgery, the dog received an intravenous injection of FA-ICG according to the provided experimental protocol (0.3 mg/kg of FA-ICG, concentration of 200 µM in PBS, containing 0.1% FA-free BSA). During surgery, intraoperative imaging was performed using the Solaris (PerkinElmer) open-air fluorescence imaging with a fluorescence channel at 800 nm (NIR channel).

### Statistical analysis

In vivo imaging data and biodistribution studies means were compared using a one-way ANOVA test followed by the Bonferroni–Dunn method for multiple mean comparison, *p*-value: *<0.05, **<0.01, ***<0.001. All statistical analyses were performed using GraphPad Prism 10.0 software.

### Ethics statement

The study was conducted according to the guidelines of the Declaration of Helsinki and approved by the Institutional Review Board (or Animal Ethics Committee) of Erasmus MC, Rotterdam, the Netherlands, under the approved work protocols SP2300047 and SP2300238, approval date 08-05-2023 and 29-02-2024 respectively, covered by the national project license CCD number AVD101002017867. Mice were housed and treated in accordance with the animal protocols approved by the Ethical Committee of the Animal Welfare Body of Erasmus Medical Center, the Netherlands. Experiments were carried out according to ARRIVE Guidelines. In the dog companion, the dog owner's informed consent was asked for, and signed approval was granted by the owner of the companion animals. Approval form of the animal owner is available at request.

## Supplementary information

Supplementary information

Supplementary Movie 1

Supplementary Movie 2

Supplementary Movie 3

## Data Availability

All data are available in the main text or the supplementary information. The figures' raw data are available at Zenodo. Additional data not reported are available upon reasonable request to the corresponding authors.
