# Author Correction: Near-infrared fatty acid molecular probe for image-guided surgery of glioblastoma

**DOI:** 10.1038/s44303-026-00185-4

**Published:** 2026-07-15

**Authors:** Meedie Ali, Pavlo Khodakivskyi, Ioannis Ntafoulis, Koen T. H. van der Kuil, Kranthi M. Panth, Arno Roos, Aleksey Yevtodiyenko, Kevin P. Francis, Zhenyu Gao, Martine L. M. Lamfers, Clemens W. G. M. Löwik, Laura Mezzanotte, Elena A. Goun

**Affiliations:** 1 Department of Radiology and Nuclear Medicine, Erasmus MC Cancer Institute, Erasums university Medical Center, Dr. Molewaterplein 40, Rotterdam, The Netherlands; 2 Department of Molecular Genetics, Erasmus MC Cancer Institute, Erasmus University Medical Center, Dr. Molewaterplein 40, Rotterdam, The Netherlands; 3 Department of Neurosurgery, Erasmus MC Cancer Institute, Brain Tumor Center, Erasmus University Medical Center, Dr. Molewaterplein 40, Rotterdam, The Netherlands; 4 Department of Chemistry, University of Missouri, 601 S College Ave, Columbia, Missouri 65211 USA; 5 Department of Neuroscience, Erasmus University Medical Center, Dr. Molewaterplein 40, Rotterdam, The Netherlands; 6 Veterinary Referral Centre Korte Akkeren, Emmastraat 29, Gouda, The Netherlands; 7 SwissLumix SARL, Batiment C, Lausanne 1015, Switzerland; 8 Department of Orthopaedic Surgery, The University of California, Los Angeles, 615 Charles E. Young Drive S, Los Angeles, California 90095 USA; 9 PerkinElmer, 68 Elm Street, Hopkinton, MA USA

**Keywords:** Cancer imaging, Fluorescent dyes, Fluorescence imaging, Brain imaging, Molecular imaging, Imaging techniques and agents

Correction to: *npj Imaging* 10.1038/s44303-025-00077-z, published online 23 June 2025

“In this article, there are amendments to the references. The original article has been corrected.”

