# Supplementary material for: Near-infrared fatty acid molecular probe for image-guided surgery of glioblastoma
Source: Npj Imaging. 2025 Jun 23;3:28. doi: 10.1038/s44303-025-00077-z (PMC12185758; doi:10.1038/s44303-025-00077-z)
Supplement: Supplementary file 1 — Supplementary information [file 44303_2025_77_MOESM1_ESM.docx]

Supplementary information

Near-Infrared Fatty Acid Molecular Probe for Image-Guided Surgery of Glioblastoma

Meedie Ali*^1,2,3^*†, Pavlo Khodakivskyi*^4^*†, Ioannis Ntafoulis*^3^*, Koen van der Kuil*^3,5^*, Kranthi M. Panth*^1,2^*, Arno Roos*^6^*, Aleksey Yevtodiyenko*^7^*, Kevin Francis*^8^*, Zhenyu Gao*^3,5^*, Martine L.M. Lamfers*^3^*, Clemens W.G.M Löwik*^1,2^*, Laura Mezzanotte*^1,2^*†*, Elena Goun*^4^*†*

**This PDF file includes:**

Supplementary text

Supplementary figures S1 to S9

Supplementary movies S1 and S3

†These authors contributed equally to this work.

**Supplementary text**

**Probe synthesis**

Materials. 1,1,2-trimethyl-1H-benzo[e]indole, 6-bromohexanoic acid, 1,4-butane sultone, 16-bromohexadecanoic acid, glutaconaldehyde dianilide hydrochloride were purchased from Sigma-Aldrich Chemie GmbH (Switzerland) and were used without purification.

Analytical HPLC was performed on a Waters ACQUITY UPLC H-Class instrument using a BEH C18 1.7 µm 2.1×50 mm column with 4-min gradient of 2% to 100% acetonitrile in water containing 0.1% formic acid. Preparative HPLC was performed on a Waters HPLC system using an XTerra Prep MS C18 OBD 5 µm 19×50 mm column with 10-min gradient of 5% to 100% acetonitrile in water containing 0.1% formic acid. NMR spectra were recorded on a Bruker Avance III HD 600 MHz instrument (14.1 T, BBFO probe). HRMS measurements were conducted at the Charles W Gehrke Proteomics Center, University of Missouri, Columbia MO, on a Thermo Scientific LTQ Orbitrap Mass Spectrometer.

**Scheme S1.** Synthetic scheme for the preparation of FA-ICG (ICG-PA) probe. *Reagents and conditions:* i) 6-bromohexanoic acid in dichlorobenzene, 120°C 16 h; ii) 1,4-butane sultone in dichlorobenzene, 120°C 20 h; iii) glutaconaldehyde dianilide hydrochloride in acetic anhydride, 60°C, 30 min; iv) 3 in pyH, 70°C, 1 h; v) HOSu, EDCI in DCM, RT, 8 h; vi) NaN_3_ in DMF, 80°C, 40 h; vii) H2, Pd/C in methanol, RT, 1h; viii) DIPEA in DMF, RT, 16h.

**ICG-CO_2_H** dye (**5**) was synthesized by previously published procedures (*99*).

**ICG-NHS** (**6**): A mixture of 5 (73 mg, 0.1 mmol), N-hydroxysuccinimide (14 mg, 0.12 mmol), EDCI (23 mg, 0.12 mmol), and DCM (5 ml) were stirred at ambient temperature for 8h. The resulting mixture was diluted with DCM (20 ml), washed with aqueous sodium bicarbonate solution (3 x 25 ml), dried over anhydrous MgSO_4_, and evaporated to give title compound **6** (76 mg, 92%) as a dark-green solid.

HRMS (ESI/QTOF): m/z calc. for C_49_H_53_N_3_O_7_S [M+H]^+^ 828.36770, found 828.36642.

**16-azidohexadecanoic acid** (**8**) was prepared by a modified procedure published before (*54*). A mixture of 16-bromohexadecanoic acid **7** (335 mg, 1 mmol), sodium azide (130 mg, 2 mmol), and DMF (10 ml) was stirred at 80°C for 40 hours. The reaction mixture was diluted with saturated aqueous ammonium chloride solution. Resulting precipitate was filtered, washed with water, dried, and crystallized in acetonitrile to give pure title compound **8** (232 mg, 78%) as a colorless solid. ^1^H NMR (600 MHz, CDCl_3_) δ 3.25 (t, *J* = 7.0 Hz, 2H), 2.35 (t, *J* = 7.5 Hz, 2H), 1.67 – 1.57 (m, 4H), 1.43 – 1.19 (m, 22H).

HRMS (ESI/QTOF): m/z calc. for C_16_H_32_N_3_O_2_ [M+H]^+^ 298.24890, found 298.24873.

**16-aminohexadecanoic acid** (**9**): A mixture of **8** (230 mg, 0.77 mmol), 10% Pd on activated carbon, and methanol (50 ml) was stirred at ambient temperature for 1 hour under hydrogen atmosphere (1 atm). The catalyst was filtered off, washed with methanol (100 ml), and combined methanol filtrate was evaporated to dryness to give title compound **9** (198 mg, 95%) as a colorless solid. ^1^H NMR (600 MHz, DMSO) δ 3.29 (m, 2H), 2.17 (t, *J* = 7.4 Hz, 2H), 1.47 (t, *J* = 7.1 Hz, 2H), 1.23 (s, 20H), 0.85 (t, *J* = 6.8 Hz, 2H).

HRMS (ESI/QTOF): m/z calc. for C_16_H_34_NO_2_ [M+H]^+^ 272.25841, found 272.25791.

**FA-ICG** (**10**): A mixture of intermediate **9** (14 mg, 0.05 mmol), ICG-NHS (41 mg, 0.05 mmol), DIPEA (13 mg, 0.1 mmol) and DMF (1 ml) was stirred at ambient temperature for 16 hours. After completion of the reaction, the resulting mixture was acidified with formic acid and the product was isolated by preparative HPLC on an RP C18 column, eluting by acetonitrile in water 0-100% over 10 min, to yield title compound **10** (17 mg, 35%) as a dark-green solid. ^1^H NMR (600 MHz, CDCl_3_) δ 8.05 (m, 2H), 7.90 (m, 4H), 7.77 (m, 1H), 7.57 (m, 2H), 7.43 (m, 3H), 7.32 (d, J = 8.8 Hz, 1H), 6.79-5.94 (br, 2H), 4.21 (m, 2H), 4.10 (m, 2H), 2.33 (m, 4H), 2.16 (m, 4H), 1.98-1.84 (m, 13H), 1.77 (m, 3H), 1.59 (m, 4H), 1.50 (m, 3H), 1.35-1.15 (m, 29H).

HRMS (ESI/QTOF): m/z calc. for C_61_H_82_N_3_O_6_S [M+H]^+^ 984.59188, found 984.59259.

NMR spectra of **8**, **9** and **10**

**
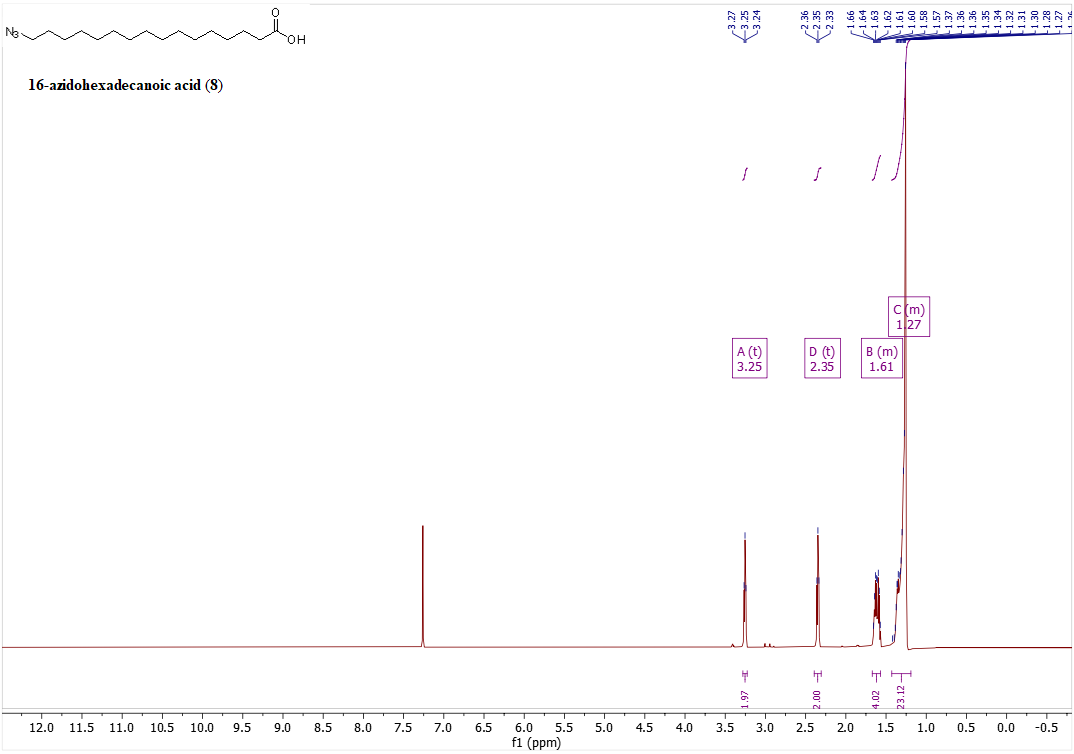
**

**
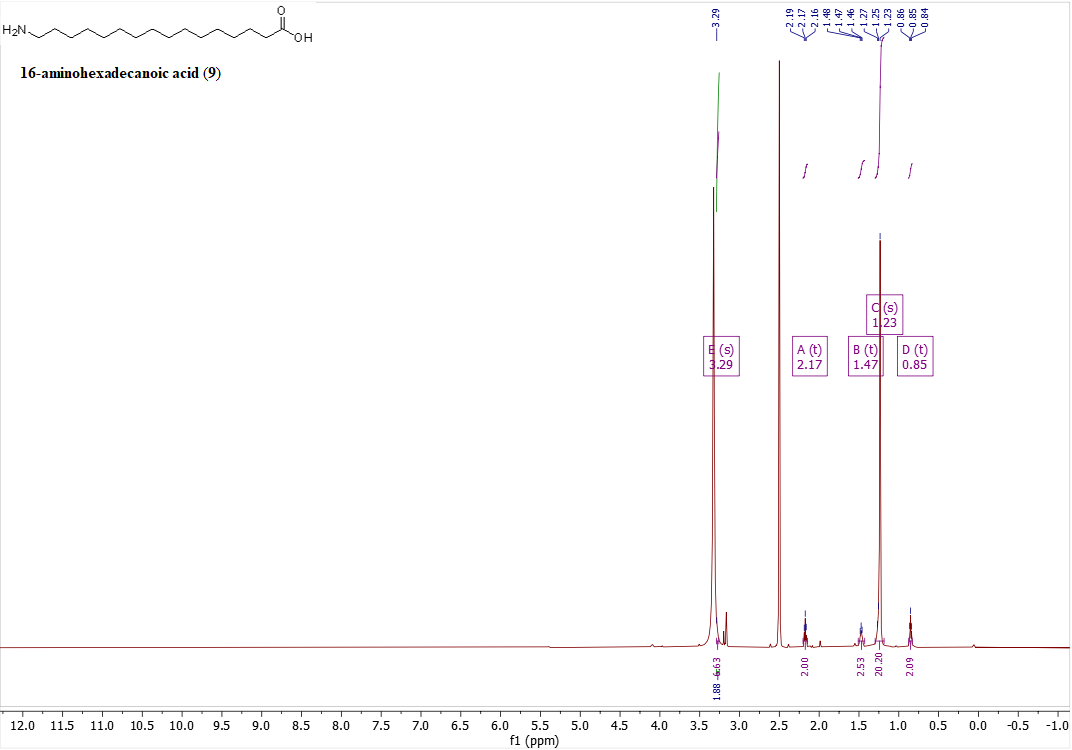
**


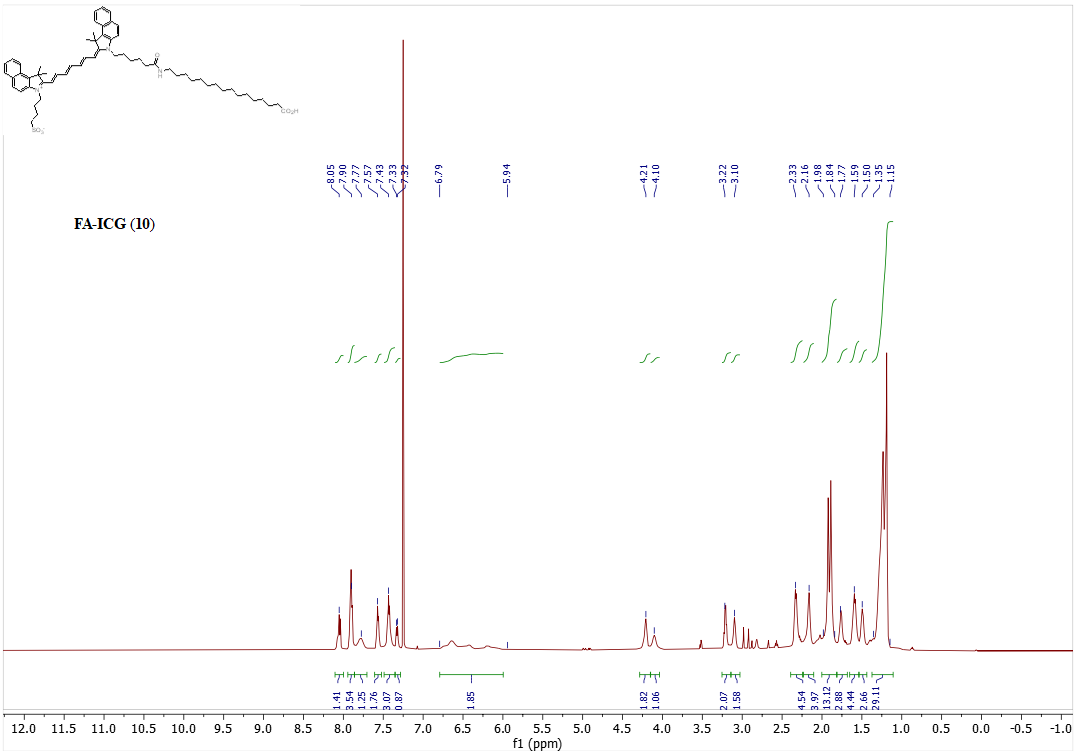


HRMS spectra of **6**, **8**, **9**, and **10**


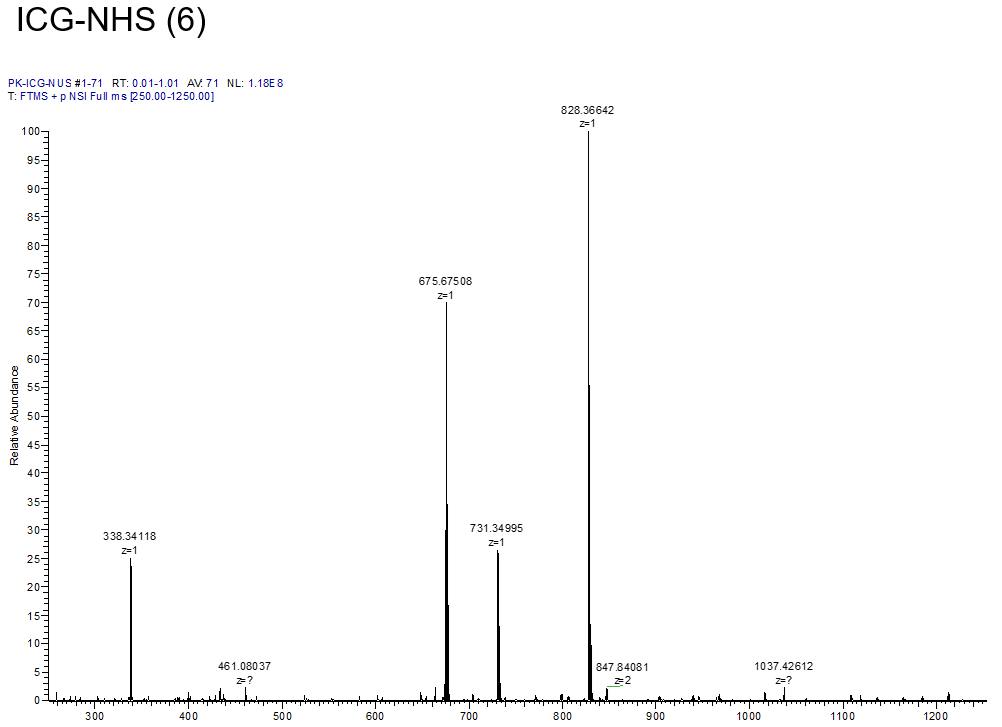


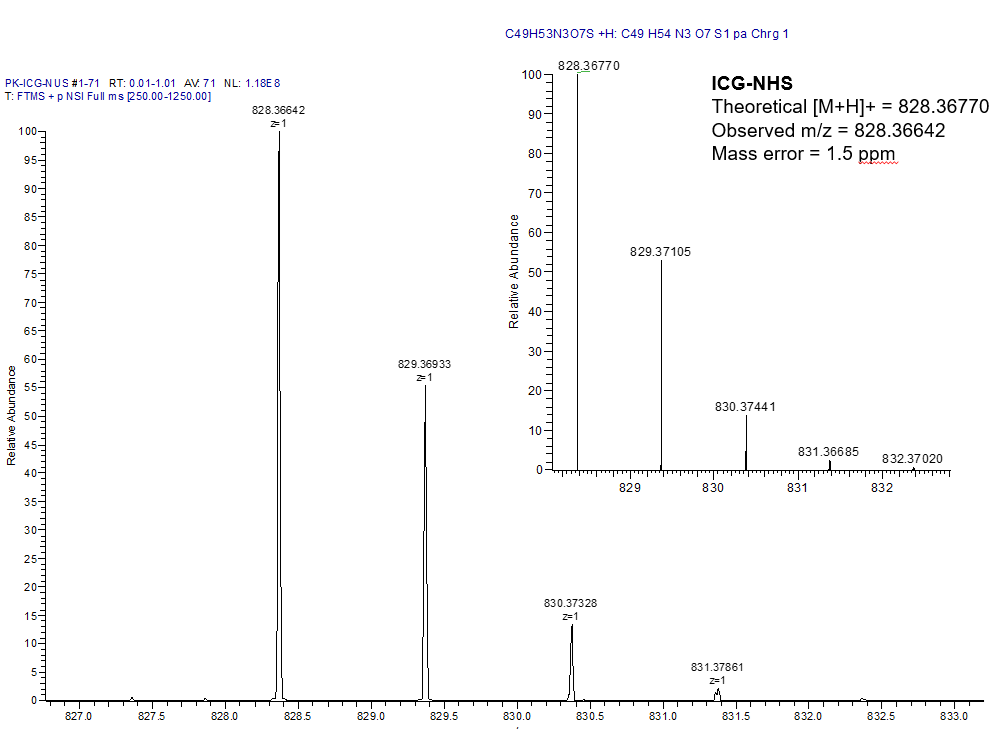


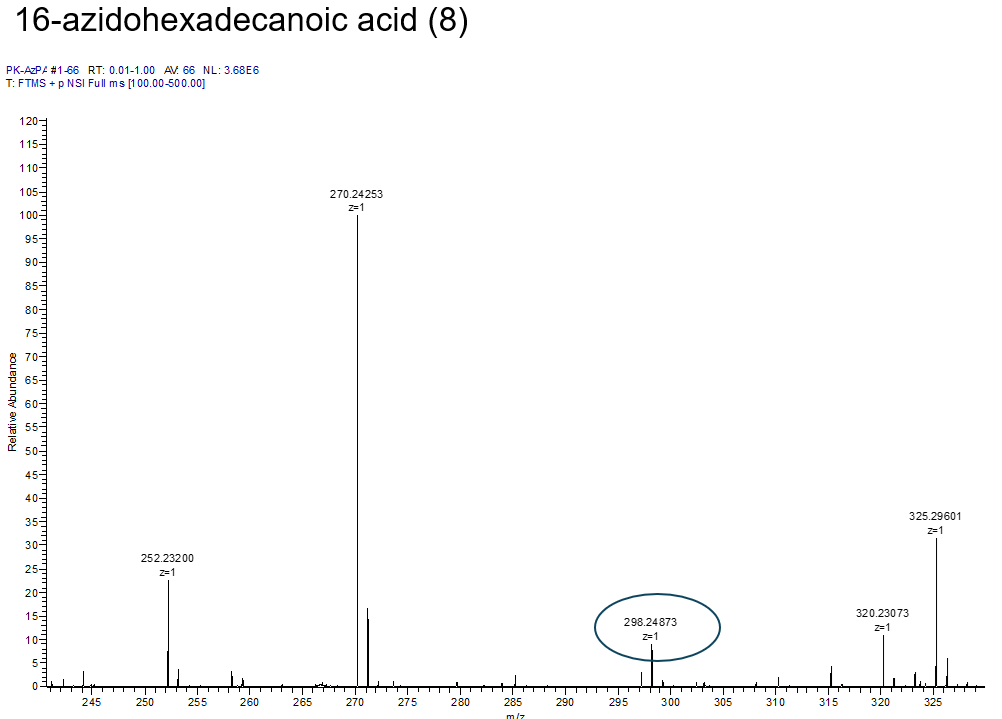


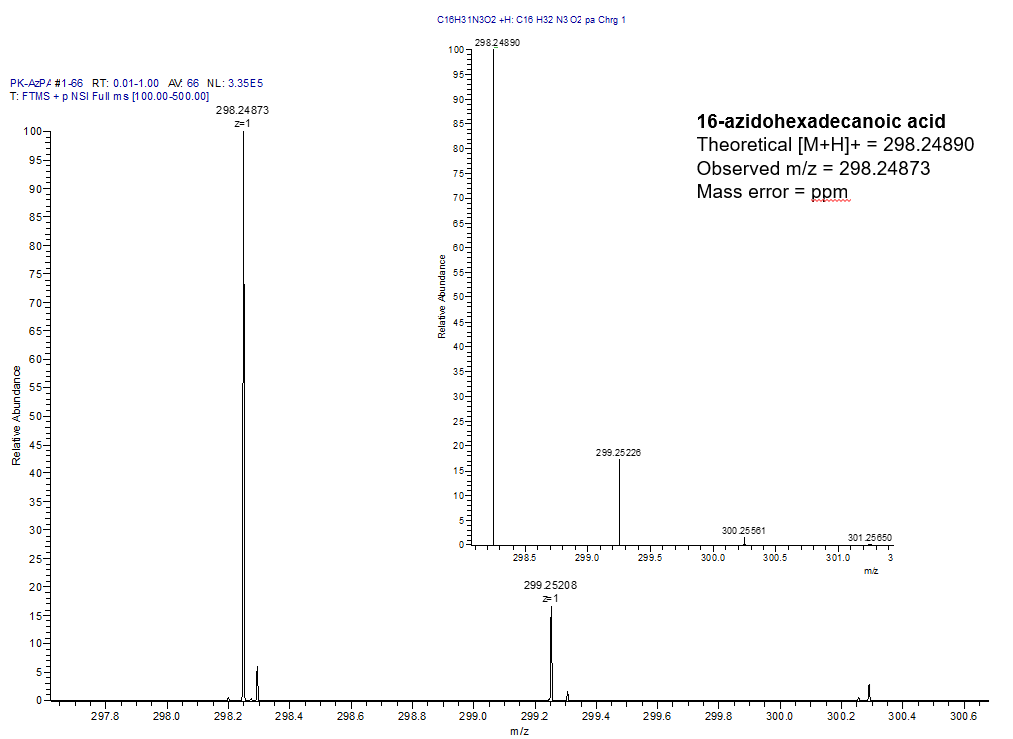


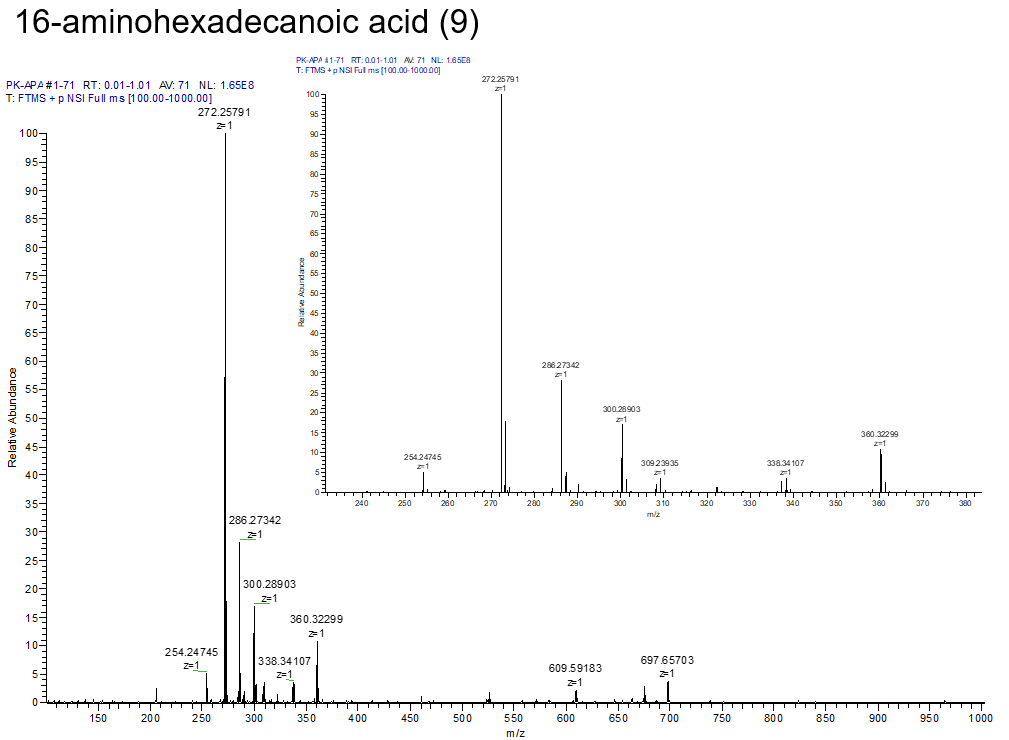


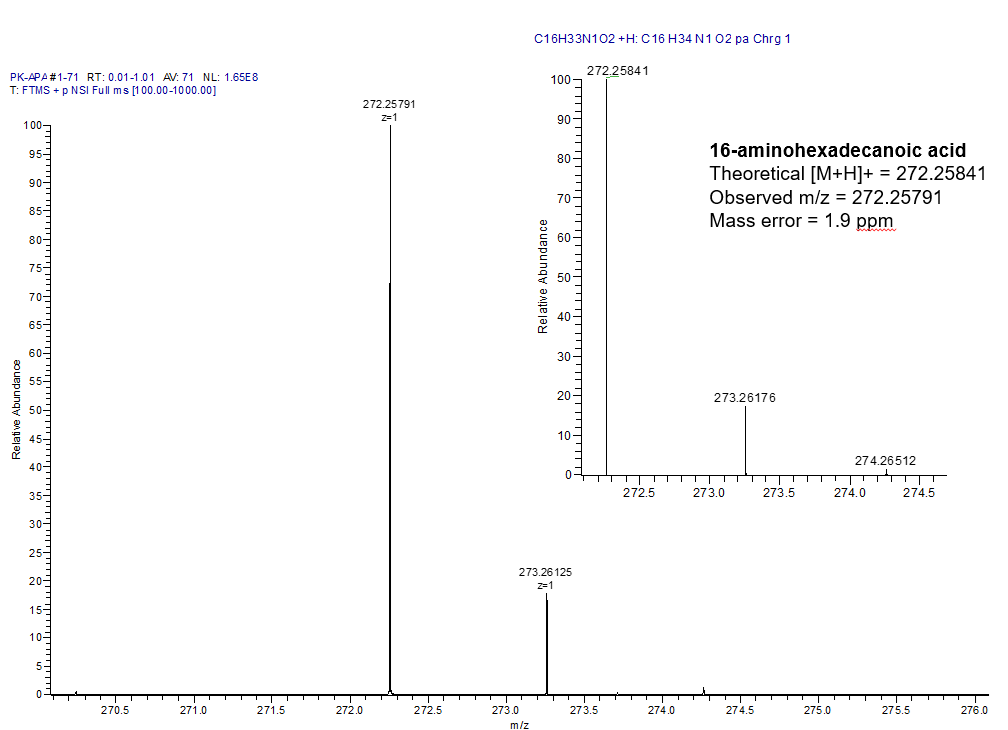


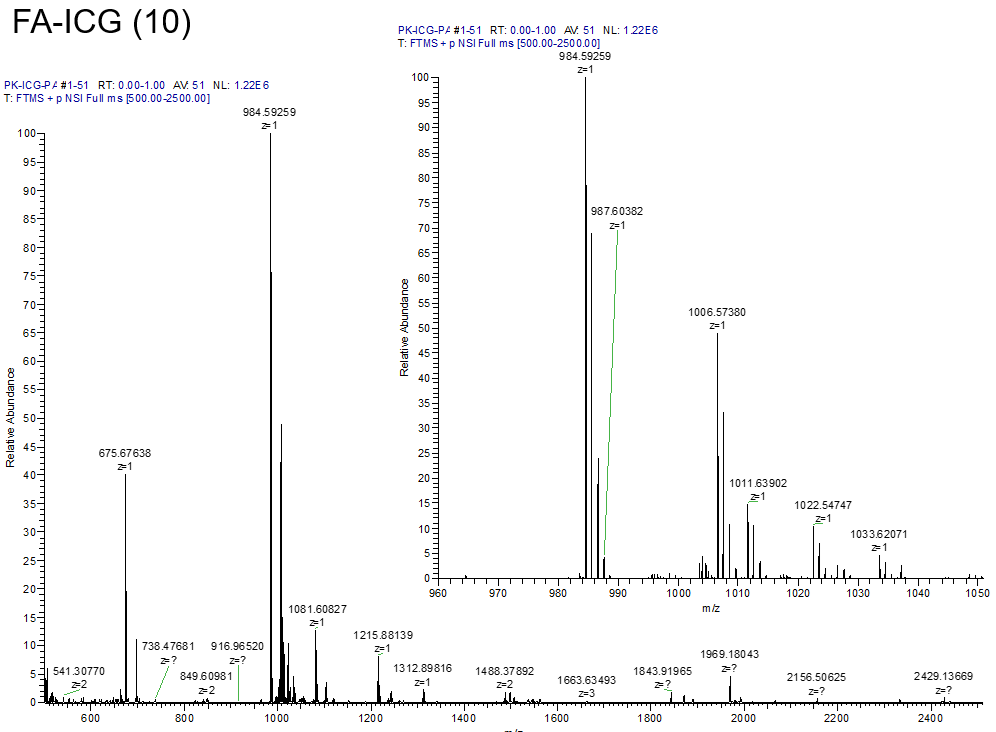


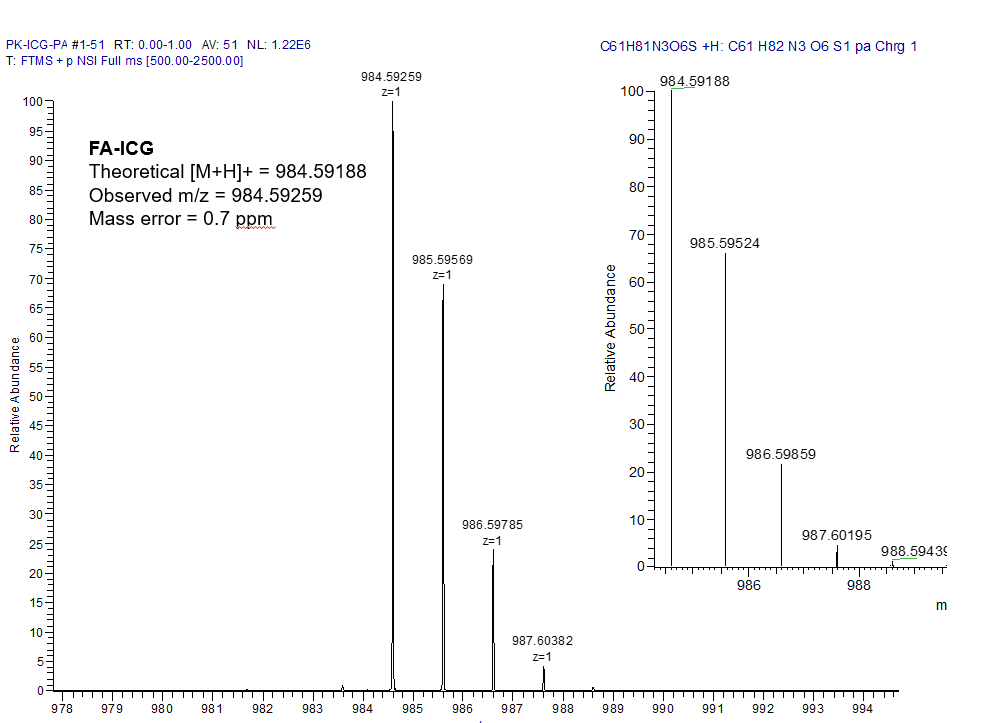


**Supplementary figures**

**
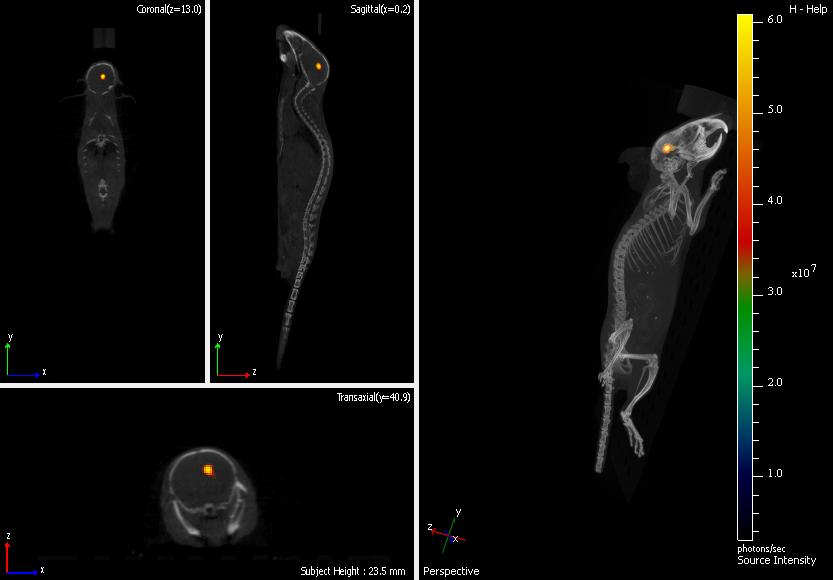
**

**Figure S1. Visualization of FA-ICG in U87-MG orthotopic glioma tumor bearing mouse using FMT-CT.** Reconstruction of NIR signal using FMT-CT fluorescence imaging at 24 hours after *i.v.* administration of FA-ICG in orthotopic tumor-bearing U87-MG-luc glioblastoma mouse. For three-dimensional reconstruction see also **Movie S1**.

**
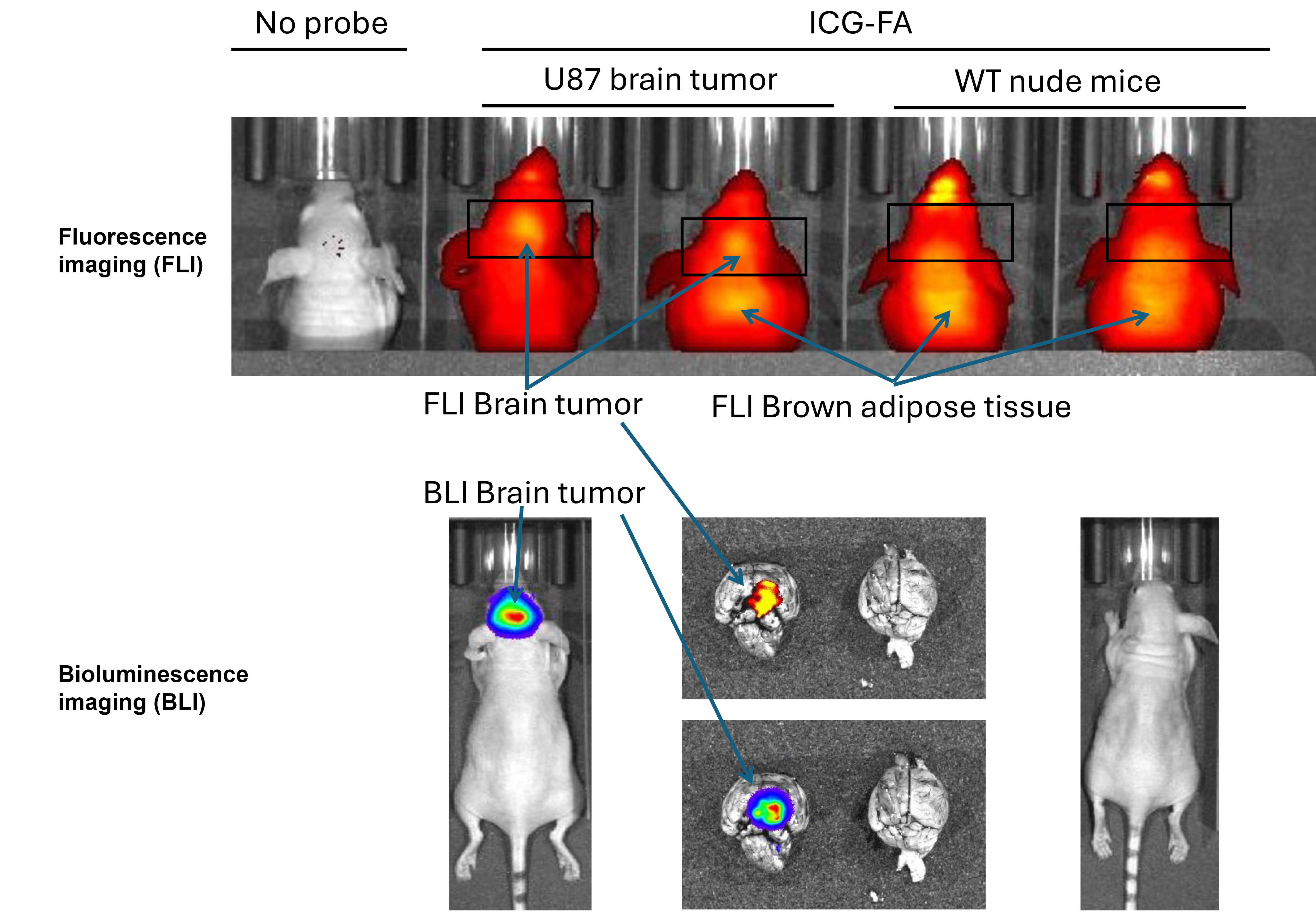
**

**Figure S2. *In -* and *ex vivo* accumulation of FA-ICG in orthotopic U87-MG tumor-bearing mouse brain.** Tumor-bearing (U87-Fluc) and non-tumor bearing (WT) nude mice were administered FA-ICG, 24 hours after injection fluorescence imaging was performed. FLI in tumor bearing mice showed accumulation in the brain tumor as well as in brown adipose tissue while in non-tumor bearing mice only adipose tissue showed accumulation. The tumor-bearing mouse was co-administered d-luciferin *i.p.*, bioluminescence- and fluorescence imaging co-localize in the U87-MG-luc brain while the healthy animal does not display luminescence nor fluorescence signal.


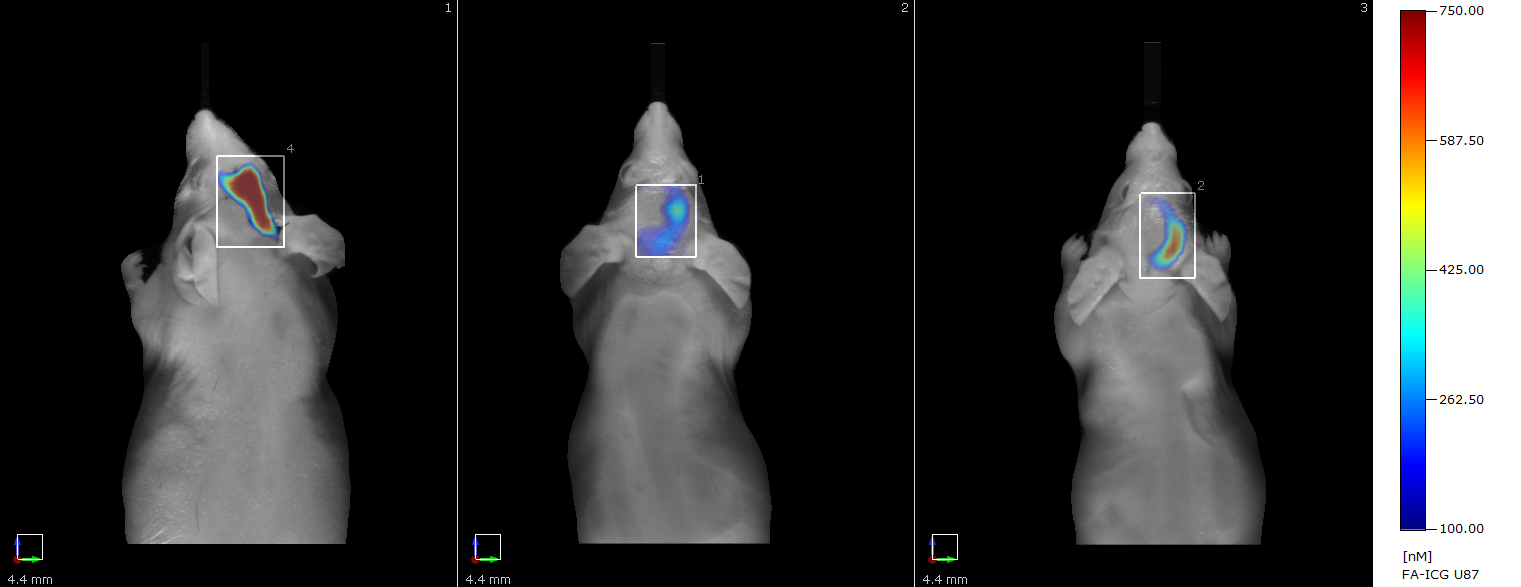


**Figure S3. *In vivo* imaging of FA-ICG in orthotopic U87-MG tumor bearing mouse (8-, 12- and 24-hours post-injection).** Fluorescence molecular tomography (FMT) of orthotopic glioblastoma tumor-bearing mice (n=1 per time point) at 8-, 12- and 24-hours post-administration of FA-ICG (from left to right: 8-, 12- and 24-hour imaging). Fluorescence signal is most intense at the right side of the head of each mouse (in accordance with the tumor inoculation site in the right cerebral hemisphere). Color bar represents fluorescence signal intensity (blue = low, to red = high).

**
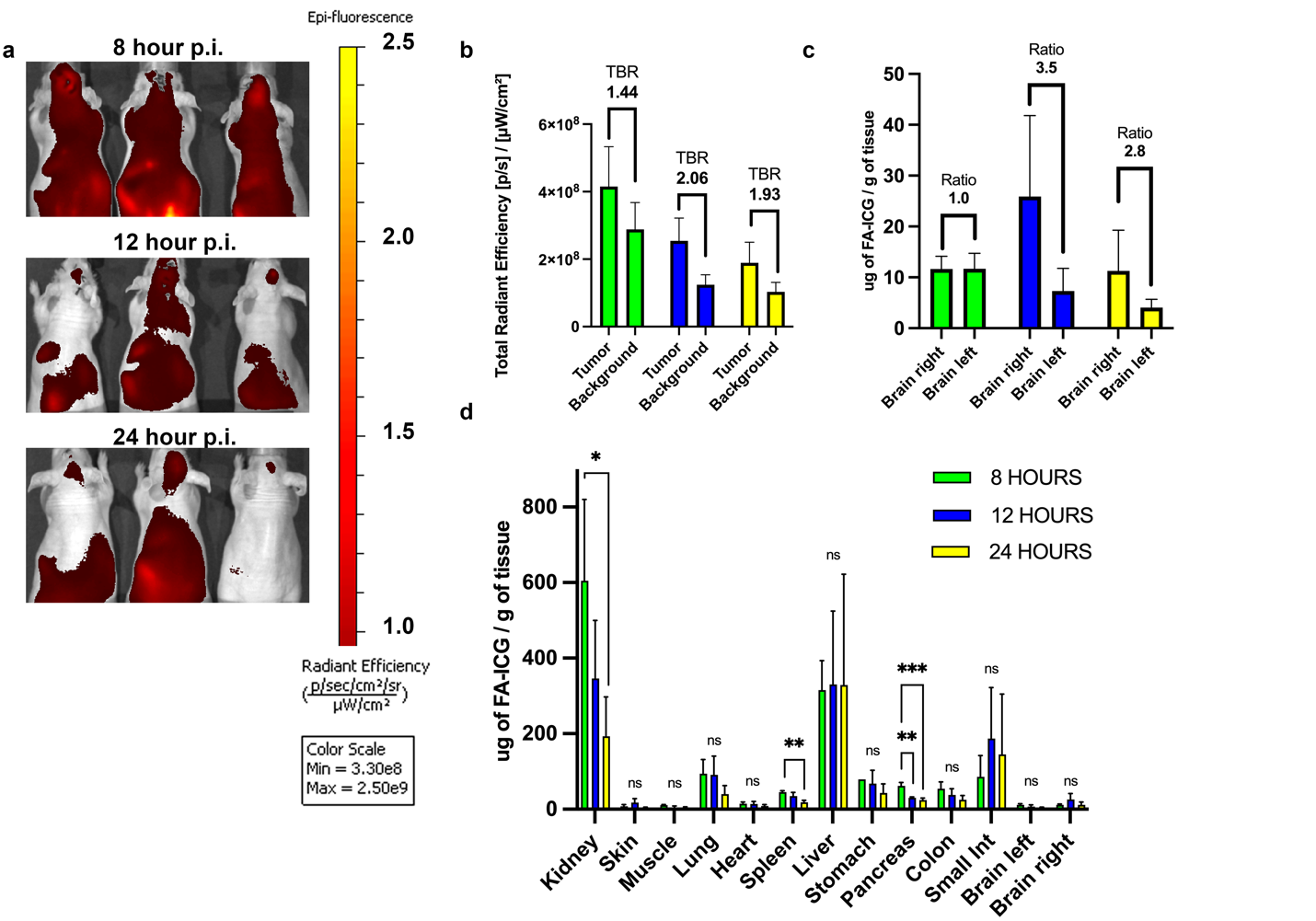
Figure S4. Imaging and quantification of FA uptake in orthotopic glioblastoma xenograft model (U87-MG). a** Whole-body fluorescence imaging was performed at three time points (8-, 12- and 24 hours) after intravenous administration of FA-ICG. Absolute fluorescent signal decreased over time, while accumulation in the brain increased. **b** Tumour-to-background ratio (TBR) in the head was demonstrated to improve over time, with 12 and 24 hrs imaging demonstrating the best TBR. **c** *Ex vivo b*iodistribution of FA-ICG probe in the brain confirmed these findings. The right (tumour-bearing hemisphere) was demonstrated to accumulate more of the probe over time when compared to the left brain hemisphere. **d** *Ex vivo* biodistribution of FA-ICG in orthotopic brain tumour mouse model. The highest signal from FA-ICG probe is observed in kidney and liver. Sample size per experimental group for experiments described is n=3. Error bars report on standard deviation. Statistical analyses were performed using a One-Way Anova test followed by Bonferroni-Dunn method for multiple mean comparison. Statistical significance was set at *p* < 0.05 (*<0.05, **<0.01, ***<0.001).

**
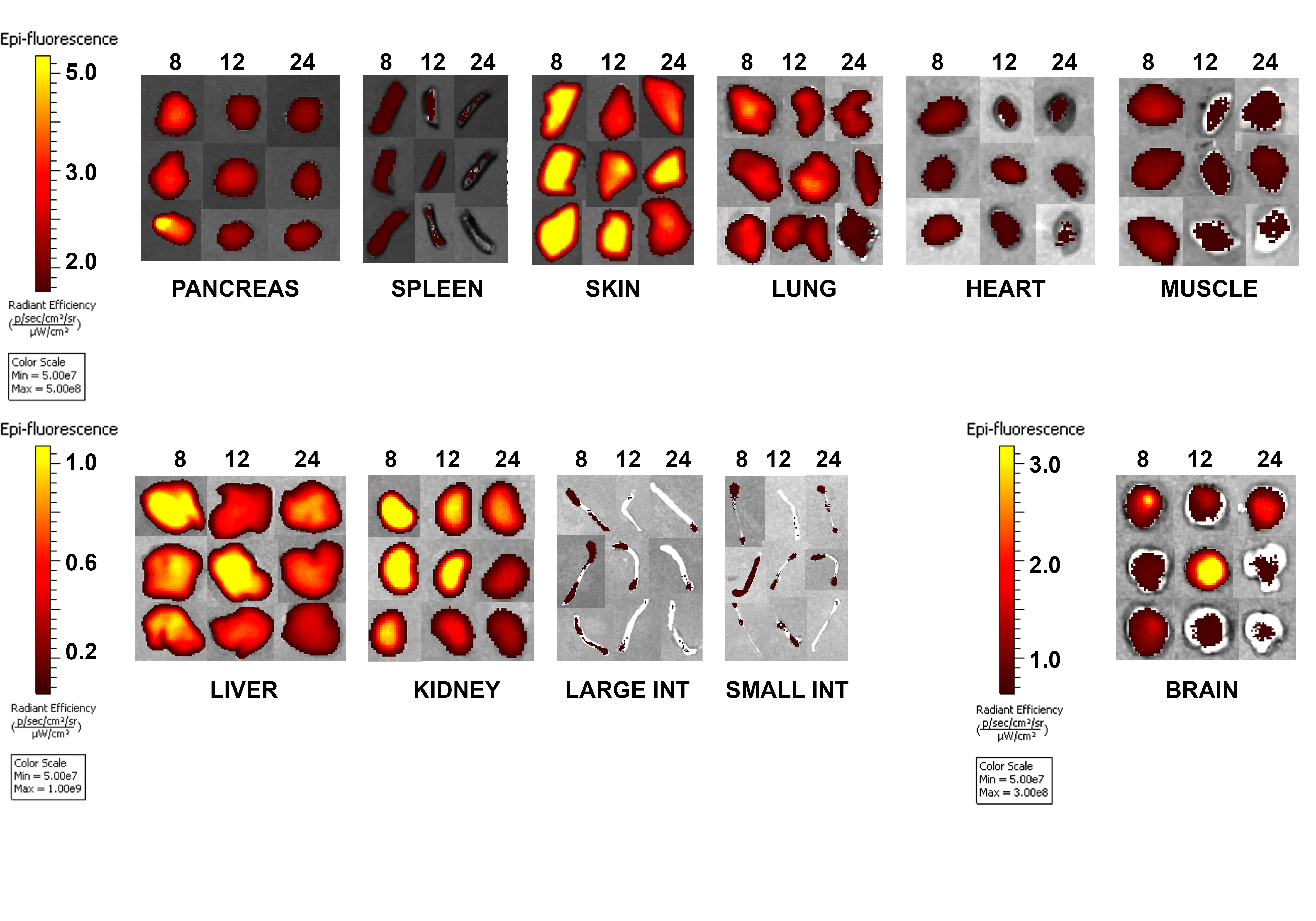
Figure S5. *Ex vivo* per organ biodistribution of FA-ICG in orthotopic U87-MG tumor-bearing mouse.** *Ex vivo* fluorescence imaging of a various pallet of organs at 8-, 12- and 24-hours post-administration of FA-ICG in orthotopic tumor bearing nude mouse. Organs are arranged in three groups according to similar range in signal intensities.

**
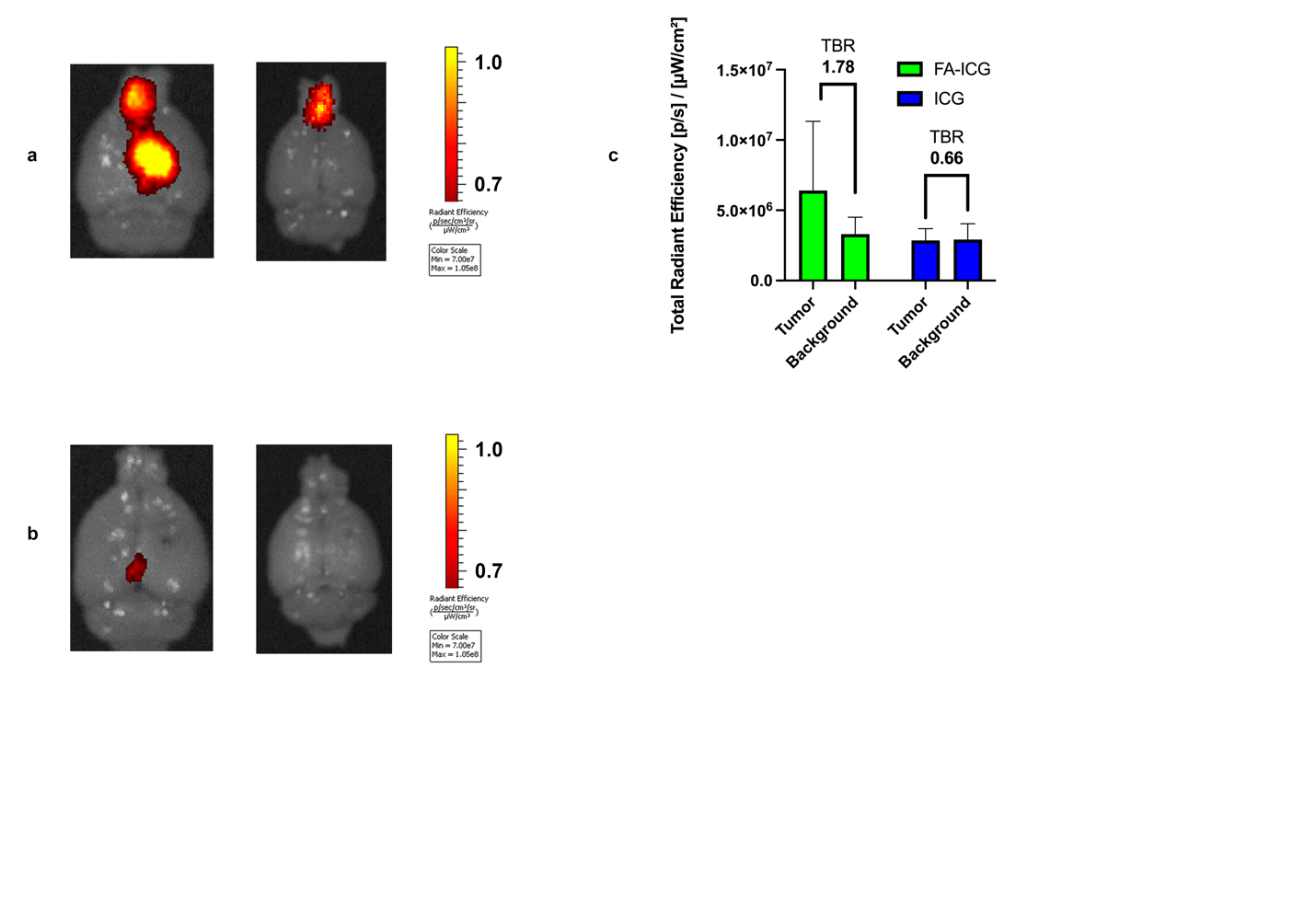
**

**Figure S6. *Ex vivo* accumulation of FA-ICG vs ICG in orthotopic U87-MG glioblastoma model.** **a-c** *Ex vivo* brain imaging of FA-ICG (**a**) and ICG (**b**) administered mice demonstrates significantly higher TBR (**c**) in FA-ICG administered mice at 8 hours post-administration.

**
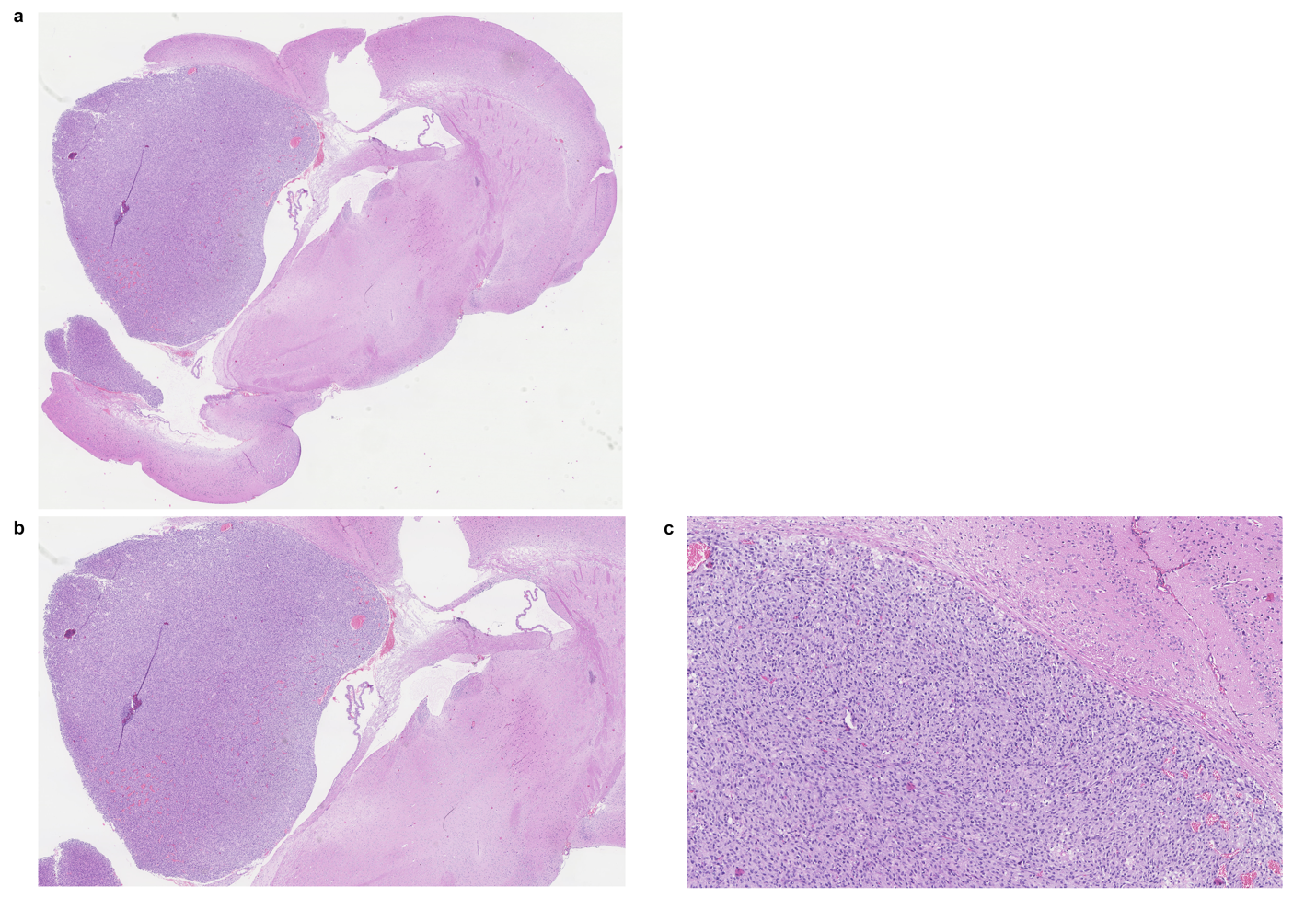

Figure S7. H&E-staining of large orthotopic U87-MG glioblastoma tumor in right hemisphere. a-c** Haematoxylin Eosin staining as representative histological confirmation of U87-MG tumor bearing mouse. Mouse sacrificed at 14 days post-inoculation of U87-MG cell line in NMRI nude mouse.

**
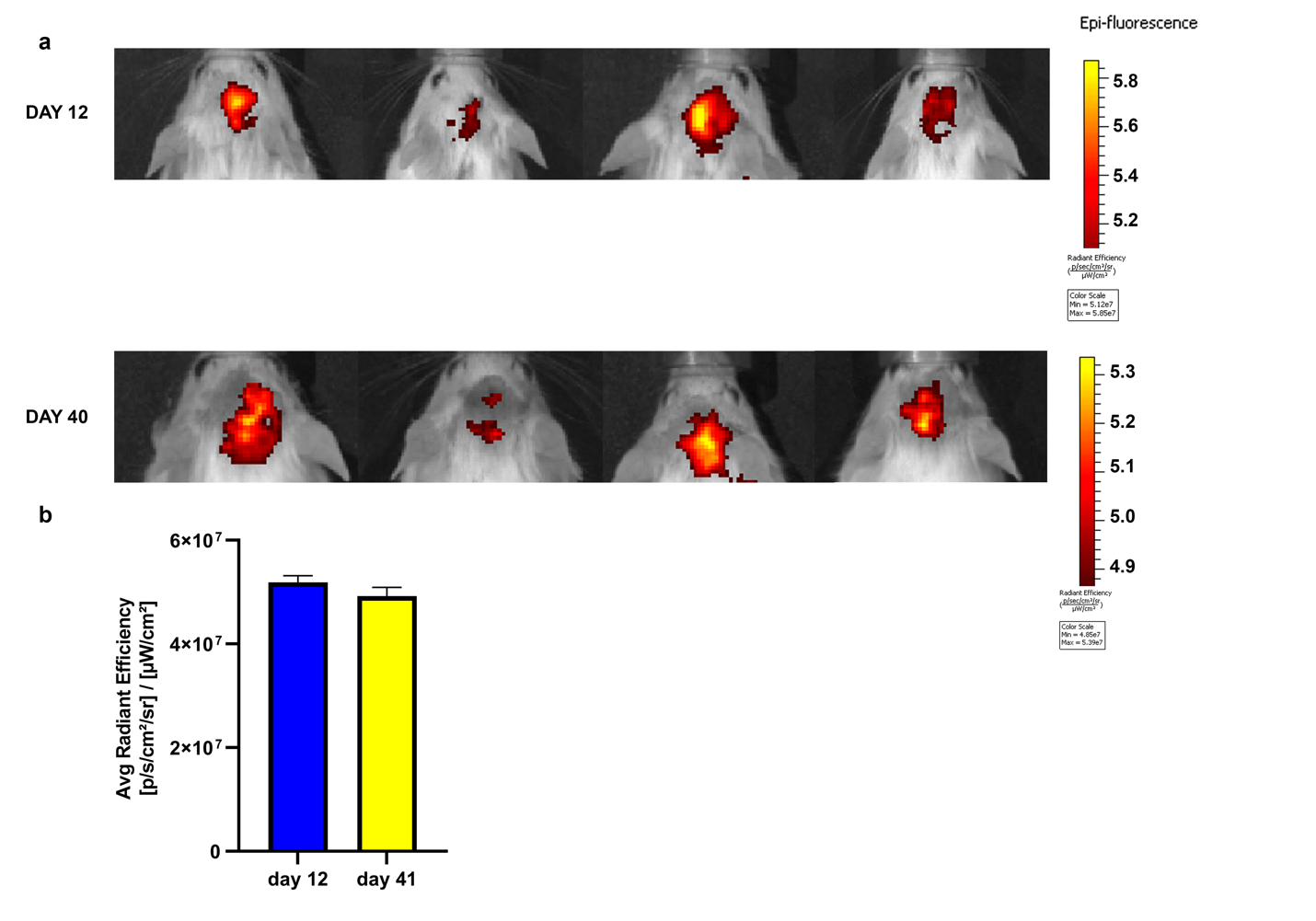
**

**Figure S8. Longitudinal fluorescence imaging of tumour growth in orthotopic patient-derived GSC glioblastoma tumour model using FA-ICG probe. (A)** Representative images of orthotopic patient-derived tumour-bearing mice (glioblastoma cell line, GS607) at 24 hours post-administration of FA-ICG at 12 and 41 days after tumour inoculation. **(B)** Signal quantification demonstrates a stable average radiant efficiency. Sample size per experimental group is n=4.


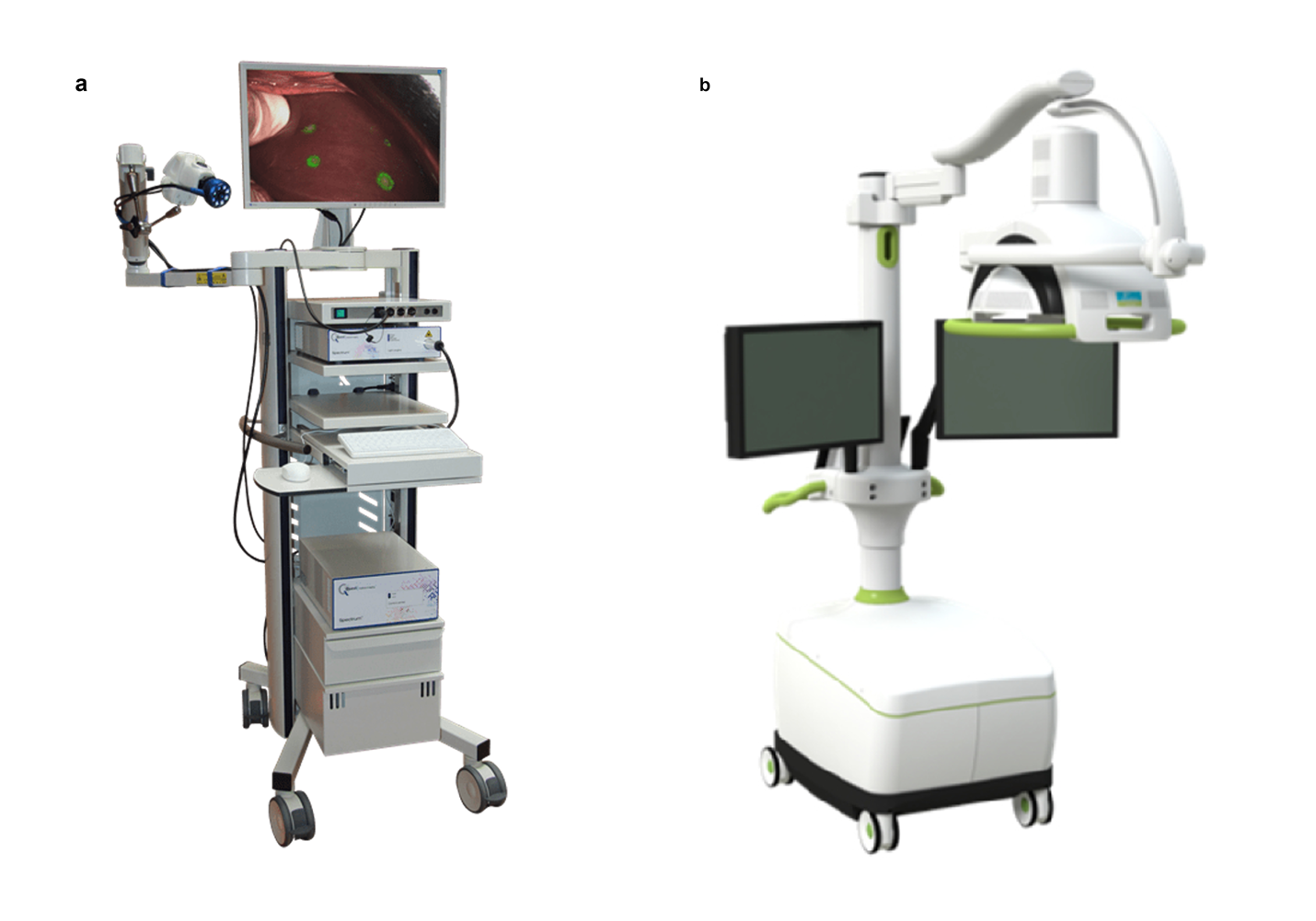


**Figure S9. Near-infrared image-guided surgery camera systems. a** QUEST Spectrum® 2 near-infrared image-guided surgery camera (QUEST Medical Imaging). **b** Solaris™ open-air fluorescence imaging camera (PerkinElmer®).

**Movie S1. *In vivo* imaging and - co-localization of FA-ICG in orthotopic glioblastoma xenograft model (U87-MG-Luc).** Three-dimensional reconstruction demonstrating co-localization of intracranial fluorescence and bioluminescence signal on FMT-CT at 24 hours after probe administration in U87-MG-Luc tumor-bearing mouse.

**Movie S2. Fluorescence-guided surgery in orthotopic U87-MG nude mouse.** Craniotomy of orthotopic glioblastoma tumor bearing mouse using fluorescence-guided surgery 8 hours after *i.v.* administration of FA-ICG or ICG. Transcutaneous, transcranial and view of the parenchyma is shown sequentially, as well as *ex vivo* close-up imaging of the brains.

**Movie S3. Fluorescence-guided surgery of canine mastocytoma.** Surgical removal of mastocytoma in a dog using fluorescence-guided surgery 10 hours after *i.v.* administration of FA-ICG.
